# Poly(lactide) Upcycling Approach through Transesterification for Stereolithography 3D Printing

**DOI:** 10.1021/acs.biomac.4c00840

**Published:** 2024-10-03

**Authors:** Silvestr Figalla, Vojtěch Jašek, Jan Fučík, Přemysl Menčík, Radek Přikryl

**Affiliations:** † Institute of Materials Chemistry, Faculty of Chemistry, Brno University of Technology, 61200 Brno, Czech Republic; ‡ Institute of Environmental Chemistry, Faculty of Chemistry, Brno University of Technology, 612 00 Brno, Czech Republic

## Abstract

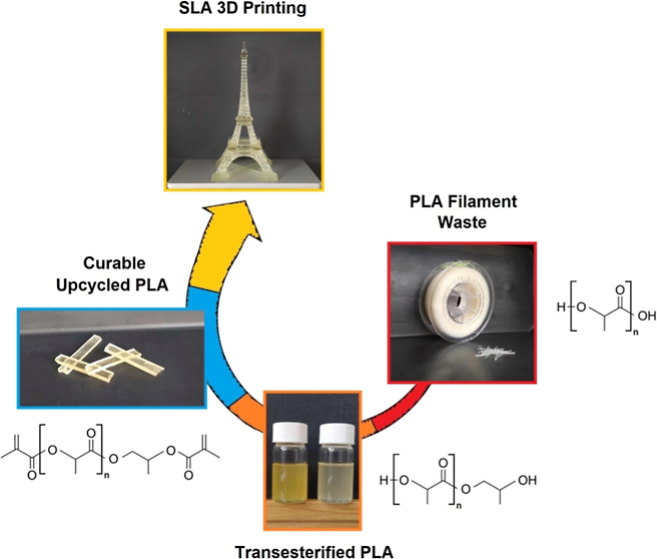

The legislature determines the recycled and waste contents in fabrication processes to ensure more sustainable production. PLA’s mechanical recycling and reuse are limited due to the performance decrease caused by thermal or hydrolytic instability. Our concept introduces an upcycling route involving PLA depolymerization using propylene glycol as a reactant, followed by the methacrylation, assuring the liquid systems’ curability provided by radical polymerization. PLA-containing curable systems were studied from a rheological and thermomechanical viewpoint. The viscosity levels varied from 33 to 3911 mPa·s at 30 °C, giving a wide capability potential. The best system reached 2240 MPa storage modulus, 164.1 °C glass-transition temperature, and 145.6 °C heat-resistant index, competitive values to commercial systems. The printability was verified for all of the systems. Eventually, our concept led to SLA resin production containing PLA waste content up to 51 wt %.

## 1 Introduction

Polylactide (PLA), as an aliphatic polyester based on renewable sources,^1^ serves numerous applications such as a matrix for medical components and devices,^2,3^ the clothing and interior fiber material,^4^ a popular alternative for 3D printing filament manufacture,^5^ or as a packaging material.^6^ The biobased origin is essential in the industrial future since material compositions tend to be determined legislatively.^7^ This factor mainly affects fiber and packaging applications due to their higher disposability. On the other hand, medicinal and 3D printing applications benefit from PLA physical–chemical properties for their processing.^8^ According to many recent investigations, the absence of adverse effects on organisms’ physiological functions ensures the role of PLA-containing systems in combination with additional additives and compounds such as cellulose acetate^46^ or ZnO^47^ in animal and human bone tissue engineering and various medicinal implants.^9,46−48^ PLA barrier properties conduce to the usage in drug delivery systems.^10^ Usually, medicinal applications using PLA are connected to 3D printing technologies due to their precision and high prototype-forming potential.^11^ From the manufacturing standpoint, the PLA glass-transition temperature (*T*_g_ around 60 °C)^12^ favors the polyester’s processability in printing filaments and its comfortable printability.^13^ Therefore, this technology can efficiently propose and form various complicated scaffolds.

PLA production evolves continually. By 2027, PLA production’s global capacity is expected to increase over five times, resulting in 2.38 million tons.^37^ The worldwide production of bioplastics involves 10.4% of manufactured PLA for various material purposes.^49^ According to industry estimates, bioplastics represent <3% of the total polymers used in medicinal devices and <1% of total market for medicinal polymers as a whole in 2021. The anticipated growth of bioplastic in the global polymer industry is a 25% increase by 2026.^50^ PLA polymer is mainly produced from corn starch or sugarcane.^38^ These sources are renewable, and PLA is considered a substance that utilizes CO_2_ for production.^39^ However, secondary products, wastes, and cheaper sources are desired to further decrease PLA manufacturing prices further. In particular, food waste was investigated as an alternative to primary sources for PLA production.^40,41^ Food and Agriculture Organization (FAO) released data uncovering that one-third of the world’s food waste, equal to 1.3 billion tons, is annually wasted.^42^ According to recent reviews, biowaste is composed of 58% food and 42% lignocellulose and plant residue sources. Since PLA is produced via fermentation, carbohydrates are a desired entry source for manufacture. Food waste includes 27% carbohydrates, and plant-based material contains an overall carbohydrate content of 44–51%.^42^ These values promise the potential incorporation of food and plant wastes into the poly(lactide) production route. PLA can be composted, but there are conditions that must be fulfilled for the successive decomposition. The environment must consist of industrial plant composting conditions at 55–70 °C.^44^ According to the available literature, PLA can be 100% recycled/composted, while PLA also exhibits significantly lower emissions (∼28 kg CO_2_) than other macromolecules such as polyethylene terephthalate (PET) (∼830 kg CO_2_).^43^

The concept of material upcycling can connect the benefits of the aforementioned reprocessing routes. Unlike the mechanically recycled system, the resulting material possesses chemically virgin character. Additionally, the synthesis consists of fewer process steps, increasing the process’s efficiency and decreasing the required expenses compared to chemical recycling.^14,15^ Polytetrahydrofuran (PTHF) is a widely used material, especially in the field of stretchable fabrics or in the polyurethane industry.^16^ This polymer is chemically recycled to form its monomer form, tetrahydrofuran. The traditional recycling approach is complicated due to PTHF-stable ether bonds and inefficient monomer reactivity equilibrium. Therefore, the upcycling approach was suggested, experimentally verified, and published.^17^ The upcycling strategy can serve as a way to obtain diverse monomers with added value in various application fields.^18^ Waste PET contains terephthalic acid and ethylene glycol, which can be obtained from PET quantitative hydrolysis and serves as virgin monomers for various purposes.^19^ The direct PLA upcycling concept was introduced considering polyester aminolysis. The published process results in *N*-lactoyl ethanolamine (*N*-LEA) formation, followed by the methacrylation of *N*-LEA. This approach uses EA in molar excess 4:1 to depolymerize PLA to obtain monomer *N*-LEA, and the curable product occurs as a solid-state powder after the methacrylation. Therefore, additional liquid comonomer 4-acryloylmorpholine (ACMO) was used for the resin preparation for stereolithography (SLA).^20^

This paper is focused on the PLA upcycling approach using propylene glycol (PG) as a short-chain diol molecule for PLA depolymerization via transesterification. The different PLA and PG molar ratios vary the chemical process, resulting in transesterified PLA oligomer (PLA Oligo) formation terminated with PG containing two hydroxyl groups. The formed PLA oligomers are modified using methacrylic anhydride (MAAH) in a one-pot reaction process, producing methacrylated PLA oligomers (PLA MMA). This process involves methacrylic acid (MAA) formation as a secondary product. Our proposed approach incorporates the distillation step after the synthesis to separate MAA from the primary product. This step ensures further potential usage of MAA and enhances process sustainability. Numerous structure verification analyses confirm the molecular composition of acquired products [^1^H nuclear magnetic resonance (NMR), electrospray ionization mass spectrometry (ESI-MS), volumetry, and Fourier transform infrared spectrometry (FTIR)]. The rheological characterization is provided for all of the produced substances. The cured PLA MMA thermomechanical properties, including measured storage modules (*E*′), glass-transition temperatures (*T*_g_), calculated heat-resistant indexes (*T*_S_), and cross-linking densities (ν_e_), are reported in the article. The produced curable PLA MMA oligomers are eventually experimentally tested for SLA.

## 2 Experimental Section

### 2.1 Materials

PLA filament (PLA 3D870), initially manufactured for FDM 3D printing, served as a postprinting scrap for syntheses. The PLA was previously used for FDM 3D printing at a working temperature of 210–215 °C. The manufacturer of SmartFil PLA filament is Smart Materials 3D (Alcalá la Real, Spain). PG used for depolymerization was obtained from Fichema Inc. (Czech Republic). Methacrylic anhydride (MAAH) was supplied by VISIOMER (China). Used catalysts titanium(IV) butoxide (reagent grade, 97%) and potassium acetate (>99%), photoinitiator BAPO [phenyl bis(2,4,6-trimethylbenzoyl)phosphine oxide, 97%], and *d*-chloroform (99.8% atom D) were purchased from Sigma-Aldrich (Germany). Genorad 26 was bought from UL Solutions (Northbrook, IL, USA) and used as a spontaneous polymerization inhibitor.

### 2.2 Structural Verification Methods

NMR was used to obtain the ^1^H spectrum to verify the structures of the synthesized molecules. The measurements were provided via a Bruker Avance III 500 MHz (Bruker, Billerica, MA, USA) with a measuring frequency of 500 MHz for ^1^H NMR at 30 °C. *d*-Chloroform (CDCl_3_) served as a solvent, and tetramethylsilane represented an internal standard. The chemical shifts (δ) are expressed in parts per million (ppm) units referenced by a solvent. Coupling constant *J* has (Hz) unit with coupling expressed as s-singlet, d-doublet, t-triplet, q-quartet, p-quintet, and m-multiplet.

ESI-MS provided structural verification of the produced molecules. The instrument used was a Bruker EVOQ LC-TQ, which used electrospray ionization. Product scan spectra were obtained by fragmentation of the precursor ions. Collision energy spread (5–20 eV) enhanced the collected MS/MS data quality. Furthermore, the obtained mass spectra correspond with their prediction by CFM-ID 4.0,^21^ which also proposed the product ion structure for the most intensive masses.

FTIR was used as a structure verification method. The FTIR spectrum served as one cross-analysis for structure verification, and also infrared spectrometry was chosen to describe the structural changes of all PLA Oligo and PLA MMA series. The instrumentation was a Bruker Tensor 27 (Billerica, MA, USA), and the applied method was attenuated total reflectance using diamond as a dispersion component. The diode laser was an irradiation source. A Michelson interferometer was used to quantify the signal. Spectra comprised 32 total scans with a measurement resolution of 4 cm^–1^.

Gel permeation chromatography (GPC) served as the curable PLA MMA molecular weight determination. The measurements were obtained via GPC (Agilent 1100, Santa Clara, CA, USA) in chloroform (CHCl_3_). The used standards were polystyrene (brand Polymer Laboratories), and their molecular weights and PDIs were 151,700 g/mol (PDI 1.02), 38,100 g/mol (PDI 1.03), 30,300 g/mol (PDI 1.02), 19,880 g/mol (PDI 1.02), 9920 g/mol (PDI 1.02), 8450 g/mol (PDI 1.03), 2970 g/mol (PDI 1.04), and 580 g/mol (PDI 1.11). The measured sample (3 mg) was solubilized in chloroform (1 mL). Thereupon, the solution was filtered through a 0.22 μm PTFE filter. The analysis parameters are as follows: mobile phase flow of 1 mL/min; column temperature of 30 °C; and used column: PLgel 5 μm MIXED-C (300 × 7.5 mm).

PLA Oligo molecular structure and characteristics were determined via a standardized hydroxyl value measurement (DIN EN ISO 4629-2).

### 2.3 Synthesis of 2-Hydroxypropyl Lactate Oligomers (PLA Oligo)

The PLA FDM filament scrap was transferred to a round-bottom flask (200 g), and various amounts of PG were poured to form a suspension. The molar ratios of PLA/PG were 1:1, 2:1, 3:1, and 4:1. The PG mass weights are listed in Table 1. As the last component, the transesterification catalyst titanium(IV) butoxide (1 mol %) was added to the mixture. The reaction solution was equipped into the oil heating bath and heated to the reflux temperature (PG boiling point is 188 °C). The system’s hydroxyl value was determined every 30 min to monitor the reaction. The transesterification lasted 3 h until the hydroxyl value remained constant. The mixture was cooled and served as a one-pot reactant for the following methacrylation. The monomer 2-hydroxypropyl lactate ester was distilled from the mixture (at 140 °C and 10 Torr) to obtain the structural confirmation via ^1^H NMR, ESI-MS, and FTIR.

**Table 1:** Results of PLA Oligo and PLA MMA Syntheses, Including Hydroxyl Values of PLA Oligo and GPC Analyses of PLA MMA Structures

system molar ratio	synthesis					volumetry (PLA Oligo)	GPC analysis (PLA MMA)		
	PLA (mol)	PG (mol)	MAAH (mol)	MAA (distilled) (mol)	yield (%)	hydroxyl value (mg KOH/g)	*M*_w_ (g/mol)	*M*_n_ (g/mol)	PDI (−)
1:1	0.00367	2.73	5.36	4.90	83.2	695.3	383	297	1.29
2:1	0.00367	1.37	2.62	2.45	82.3	463.8	491	378	1.30
3:1	0.00367	0.91	1.89	1.67	82.5	358.6	615	449	1.37
4:1	0.00367	0.68	1.07	1.00	81.6	233.5	858	617	1.39

^1^H NMR (500 MHz, chloroform-*d*): δ 4.22–4.14 (m, 1H), 4.11–3.98 (m, 1H), 3.90 (ddd, *J* = 7.8, 6.4, 3.1 Hz, 1H), 3.61 (dd, *J* = 11.1, 3.1 Hz, 1H), 3.39 (dd, *J* = 11.1, 7.7 Hz, 1H), 1.48–1.36 (m, 3H), 1.30–1.19 (m, 3H).

ESI-MS fragmentation spectrum (C_6_H_12_O_4_): spectrum calcd [M – H_2_O]^+^ 131.07 *m*/*z*; found 131.10 *m*/*z*.

FTIR spectrum absorption wavenumber intervals: O–H stretch. 3550–3200 cm^–1^, the C–H stretch = 3000–2840 cm^–1^, the C=O (ester) stretch = 1750–1735 cm^–1^, and the C–O (ester) stretch = 1210–1163 cm^–1^.

### 2.4 Synthesis of Methacrylated 2-Hydroxypropyl Lactate Oligomers (PLA MMA)

Methacrylation followed PLA Oligo synthesis. The synthesized PLA Oligo batch was homogenized with MAAH (the molar ratio PLA Oligo/MAAH was 1:2.1 applied for all systems). The particular MAAH reaction weight components are summarized in Table 1. The methacrylation catalyst potassium acetate (3 mol %) was added to the mixture prior to the solution’s heating up. The reaction was performed at 70 °C in the same oil heating bath as that for the previous transesterification. Based on the previously published results,^22^ the reaction time was 24 h. The crude product solution was distilled under reduced pressure to remove the formed secondary product, MAA. The distillation conditions were 145 °C and 8 Torr. The quantitative amount of MAA was distilled based on the total weight (displayed in Table 1), which corresponded with the measured mixtures’ acid values (<2 mg KOH/g). The catalysts’ extraction was the last purification step. Since both catalysts, titanium(IV) butoxide and potassium acetate, are well soluble in water, the reaction system was extracted with pure water twice. The residual water was separated from pure products by distillation. The final PLA MMA yields are summarized in Table 1. The monomer PLA MMA was synthesized similarly. The distilled and structure-characterized PLA Oligo monomer was used as a reactant for the methacrylation. This separate reaction was performed to obtain a pure monomer PLA MMA monomer form, which could be cross-analyzed.

^1^H NMR (500 MHz, chloroform-*d*): δ 5.88 (dq, *J* = 4.2, 1.1 Hz, 3H), 5.79 (p, *J* = 1.5 Hz, 1H), 5.28 (q, *J* = 6.9 Hz, 1H), 5.03 (qt, *J* = 7.0, 5.0 Hz, 1H), 4.46 (dd, *J* = 11.9, 4.9 Hz, 1H), 4.20 (dd, *J* = 11.9, 5.1 Hz, 1H), 1.95 (dt, *J* = 3.3, 1.1 Hz, 6H), 1.45 (d, *J* = 6.9 Hz, 3H), 1.40 (d, *J* = 6.9 Hz, 3H).

ESI-MS fragmentation spectrum (C_14_H_20_O_6_Na): spectrum calcd [M + Na]^+^ 307.30 *m*/*z*, found 306.90 *m*/*z*.

FTIR spectrum absorption wavenumber intervals: C–H stretch. 3000–2840 cm^–1^, C=O (ester) stretch. 1750–1735 cm^–1^, C=C stretch. 1662–1626 cm^–1^, C–O (ester) stretch. 1210–1163 cm^–1^, C=C bend. 840–790 cm^–1^.

### 2.5 Characterization Methods

PLA Oligo and PLA MMA systems were monitored by a Brookfield RVDV-II + PX rotational viscometer to describe the flow profile of every system containing different reactant ratios. The apparent viscosity dependency on the temperature was measured and rearranged to obtain Arrhenius parameters essential for the rheological description.^23^ The measurements used a Peltier platform and cone–plate geometry (40 mm with a 2° angle). The method was set as follows: shear rate of 100 s^–1^ and temperature gradient of 25–60 °C. The applied sample volume was 500 μL. The linearized interpretation of Arrhenius’s plot is formulated as follows
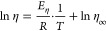
1where η is the apparent viscosity (Pa·s), η_∞_ refers to the pre-exponential factor (Pa·s), *E*_η_ is the flow activation energy (J/mol), *R* represents the universal gas constant (J/(mol·K)), and *T* refers to the thermodynamic temperature (K). The apparent viscosity (η_app_) dependency on the shear rate was investigated to verify the η_app_ constant value with a shear rate increase. This factor generally confirms the absence of polymer structure in the mixture since polymer solutions of PLA express the pseudoplastic behavior.^24^ This rheological characterization method was set for a 1–1000 s^–1^ shear rate interval.

We performed the gel content measurements for PLA MMA RESIN’s degree of cure analysis. The process is described in the ASTM D 2765-16. The sample was extracted from the Soxhlet extractor and dried until constant weight, and then the gel content was calculated according to the following equation^20^
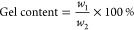
2where *w*_1_ (g) stands for the weight of the sample after the extraction and *w*_2_ (g) represents the weight of the sample before the extraction.

The thermomechanical properties were investigated by DMA 2980 from (TA Instruments, New Castle, DE, USA). The testing specimens were prepared from all PLA MMA-produced curable systems without any additional diluent. BAPO (1% w/w) was added, and precursors were cured for 30 min (405 nm LED and 8 mW·cm^–3^ of emission power). Tested objects had parameters: 60 × 10 × 2 mm. Objects were applied into a dual-cantilever attachment, and the parameters of applied deformation were 10 μm amplitude, 1 Hz frequency. The temperature increase was from 30 to 200 °C with an increase rate of 3 °C/min. The storage modulus, loss modulus, and tan δ values were obtained directly from DMA analysis. The cross-linking density ν_e_ was also calculated for all tested systems to provide information regarding the thermoset molecular structure. The equation for ν_e_ calculation stands as follows^25^
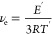
3where ν_e_ is the cross-linking density (mol/m^3^), *E*′ refers to storage modulus in the rubbery plateau region (*E*′ at *T*_g_ + 40 °C) (Pa), *R* is the gas constant (J/(mol·K)), and *T*′ stands for the thermodynamic temperature in the rubbery plateau region (at *T*_g_ + 40 °C) (K).

Thermogravimetric analysis (TGA) provided information regarding the thermal stability of the cured PLA MMA systems. Since the PLA oligomer content is variable in every cured resin, the system’s stability at increased temperatures is an essential property. The samples for TGA were obtained from the specimens for DMA analysis. Therefore, the curing process is similar to that of preparing DMA test subjects. TGA was performed on a TGA Q500 from TA Instruments (New Castle, DE, USA). The sample (10–15 mg) degradation process was monitored via heating conditions: equilibration at 40 °C, heating to 500 °C at a heating rate of 10 °C·min^–1^ under N_2_ (60 mL·min^–1^), and heating to 550 °C at a heating rate of 10 °C·min^–1^ under an air atmosphere (60 mL·min^–1^) (last step applied for cleaning purposes). TGA provided data regarding the weight decrease (and its derivation), which depend on the temperature ramp. Additionally, the equation summarizing the system’s heat resistance and defining the parameter heat-resistant index (*T*_S_) was applied to obtain the systems’ characteristics. The equation stands as follows^23^
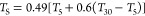
4where *T*_S_ represents the heat-resistant index (°C), *T*_5_ is the temperature at 5% of total weight loss (°C), and *T*_30_ is the temperature at 30% of total weight loss (°C).

### 2.6 SLA 3D Printing of PLA MMA

The curable mixture experimentally tested for printability consisted of the produced PLA MMA synthesized in different molar ratios with PG. Prior to the printing, 1 wt % BAPO (photoinitiator) was added to the system. The exposure time for the 3D printing process (*t*_print_) was set at 10 s to ensure the synthesized mixture’s quantitative degree of cure (405 nm LED and 8 mW·cm^–3^ of emission power). The cured layer formed on the 3D printer was 50 μm. The raft was printed to enhance attachment to the 3D printer platform and was exposed for 3*t*_print_. Then, the following 3 layers were exposed for 2*t*_print_ and the remaining for *t*_print_. The 3D printer PRUSA SL1 was used for the SLA printing experiments.

We performed additional 3D printing of testing specimens for the flexural test. The printing varied from the originally printed prototypes due to the worse printability of the PLA MMA 4:1 system. The PLA MMA 4:1 system exhibited electromagnetic irradiation molecular absorption in a similar wavelength interval as the photoinitiator BAPO. Therefore, the printing times were increased: the first 5 layers were printed for 60 s. The rest of the layers were printed for 50 s. After the printing, the specimens were washed with isopropanol and exhibited additional irradiation for 30 min. The mechanical flexural tests were performed according to standard ISO 178. The measuring parameters are as follows: loading cell with 500 N and the test speed was 10 mm/min.

## 3 Results and Discussion

### 3.1 PLA-Upcycled Oligomer Syntheses

**Figure 1:**
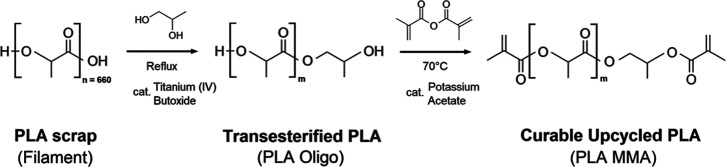
Reaction scheme of proposed PLA upcycling. PLA Oligo is the product of PLA scrap catalytic transesterification, and PLA MMA is the curable structure suitable for SLA 3D printing. Value *n* refers to calculated lactic acid units based on measured PLA number-average molecular weight.

**Figure 2:**
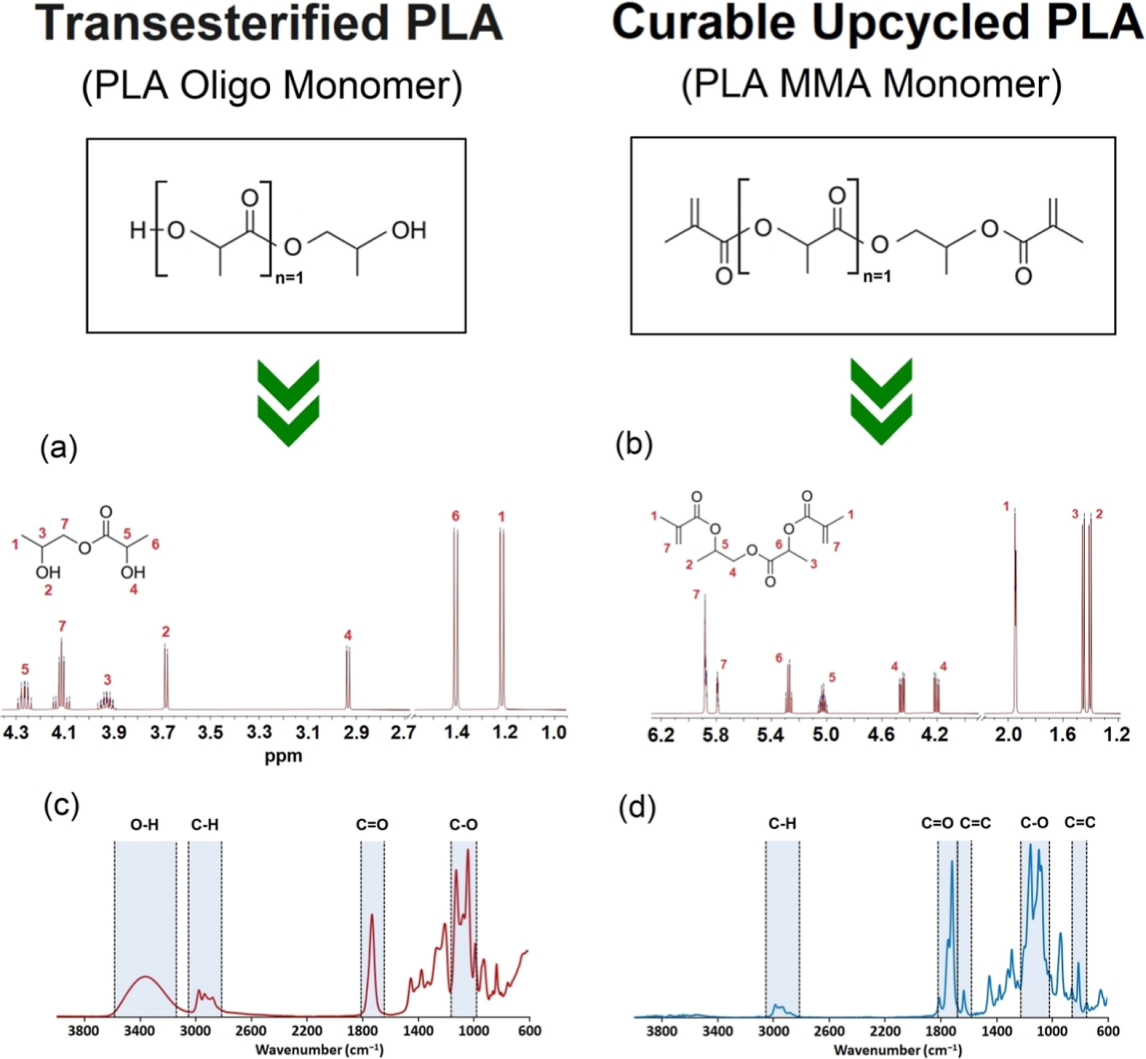
ESI-MS and FTIR spectra of synthesized PLA Oligo and PLA MMA monomers. (a) ^1^H NMR of 2-hydroxypropyl 2-hydroxypropanoate; (b) ^1^H NMR of 2-hydroxypropyl 2-hydroxypropanoate methacrylate; (c) FTIR of 2-hydroxypropyl 2-hydroxypropanoate; and (d) FTIR of 2-hydroxypropyl 2-hydroxypropanoate methacrylate.

The catalytic transesterification of PLA filament scrap was the first step to obtain a liquid and an appropriate template for the following modification via methacrylation. The PLA 3D printing material can be a leftover source suitable for upcycling regardless of its molecular weight. Since the transesterification results in complete depolymerization, the starting parameters of the used PLA are not a limitation of the process.^45^ Also, when SLA 3D printing resin is considered as the end of upcycling, the liquid state of PLA oligomers is essential for an efficient printable process. The following methacrylation ensures the presence of radically polymerizable groups in the PLA oligomer structure. The PLA oligomer functionality is a crucial parameter before the methacrylation. Methacrylic anhydride reactant weight is calculated according to the PLA Oligo length (expressed by hydroxyl value). The functionality of the PLA Oligo mixture is two hydroxyl groups per every molecule due to the nature of the depolymerization reaction (see Figure 1). The functionality of PLA Oligo was verified by cross-analysis (Figures 2 and S1). The results of PLA Oligo and PLA MMA synthesis containing reactant contents, yield values, hydroxyl values, and PLA MMA GPC-measured parameters are summarized in Table 1. The determined number-average molecular weight of used PLA scrap was 59,400 g/mol (by the described GPC method); therefore, in Table 1, the molar amount is written. The set ratios 1:1, 2:1, 3:1, and 4:1 are calculated according to the PLA monomer unit, lactic acid.

The synthesis of pure PLA Oligo monomer (2-hydroxypropyl 2-hydroxypropanoate) followed by the synthesis of monomer PLA MMA (2-hydroxypropyl 2-hydroxypropanoate methacrylate) was performed to obtain structural verification of the produced systems. Since the PLA Oligo syntheses were performed in different molar ratios and the PLA Oligo batch was immediately used as a reactant for methacrylation, we synthesized PLA Oligo monomer similarly to the 1:1 PLA Oligo synthesis, and the ester product was distilled to obtain a pure monomer form suitable for structure verification cross-analysis. ^1^H NMR provides the proposed product structure confirmation (see a, b) in connection with FTIR results (see Figure 2c,d) and ESI-MS (Supporting Information). The ESI-MS method verified for PLA Oligo monomer the molecular precursor [M – H_2_O]^+^ due to hypothetical [M – H]^+^ instability. However, the CFM-ID 4.0 spectrum projection verifies the potential instability of the precursor.^21^

Similarly, the main ESI-MS PLA MMA obtained precursor was [M + Na]^+^, which is also projected as a primary stable molecular precursor for this type of structure.^21^ The Na in a molecular precursor is located due to the used mixture of formic acid and sodium salt of formic acid (as a buffer) used for enhanced ionization for the ESI-MS-QQQ method. Although these compounds are used primarily for better ionization via electrospray, some analyzed compounds may incorporate Na for precursor stability. This scenario can be only projected by cited CFM-ID 4.0 since methacrylated 2-hydroxypropyl lactate cannot be found in the literature due to its novelty. FTIR spectra verified the presence of the particular functional groups contained in the analyzed molecules.

The calculated number-average molecular weights of PLA Oligo, and the measured hydroxyl values with the PLA MMA functionalities, are summarized in Table 2. The calculated lengths of PLA Oligo correspond with the average addition of lactic acid monomer to the longer oligomers depolymerized in varying molar amounts of PG. The obtained hydroxyl values for PLA MMA uncovered the quantitative methacrylation of all hydroxyl functional groups within PLA Oligo structures. After the calculation, the average functionality was determined as 1.99 (adjusted) for all synthesized PLA MMA systems. This outcome was also confirmed by ^1^H NMR, ESI-MS, and FTIR methods.

**Table 2:** Results of PLA Oligo and PLA MMA Volumetry Including the Calculated Molecular Weight (Number Average) of PLA Oligo and the Hydroxyl Value of PLA MMA for the Functionality Calculation

system molar ratio (PLA/PG)	calculated molecular weight (PLA Oligo)	hydroxyl value (PLA MMA)		PLA MMA RESIN
	*M*_n_ (g/mol)	hydroxyl value (mg KOH/g)	calculated functionality (−)	gel content (%)
1:1	161.4	2.9	1.99	89.4
2:1	241.9	2.4	1.99	90.4
3:1	312.9	2.0	1.99	90.3
4:1	480.5	1.4	1.99	91.4

**Figure 3:**
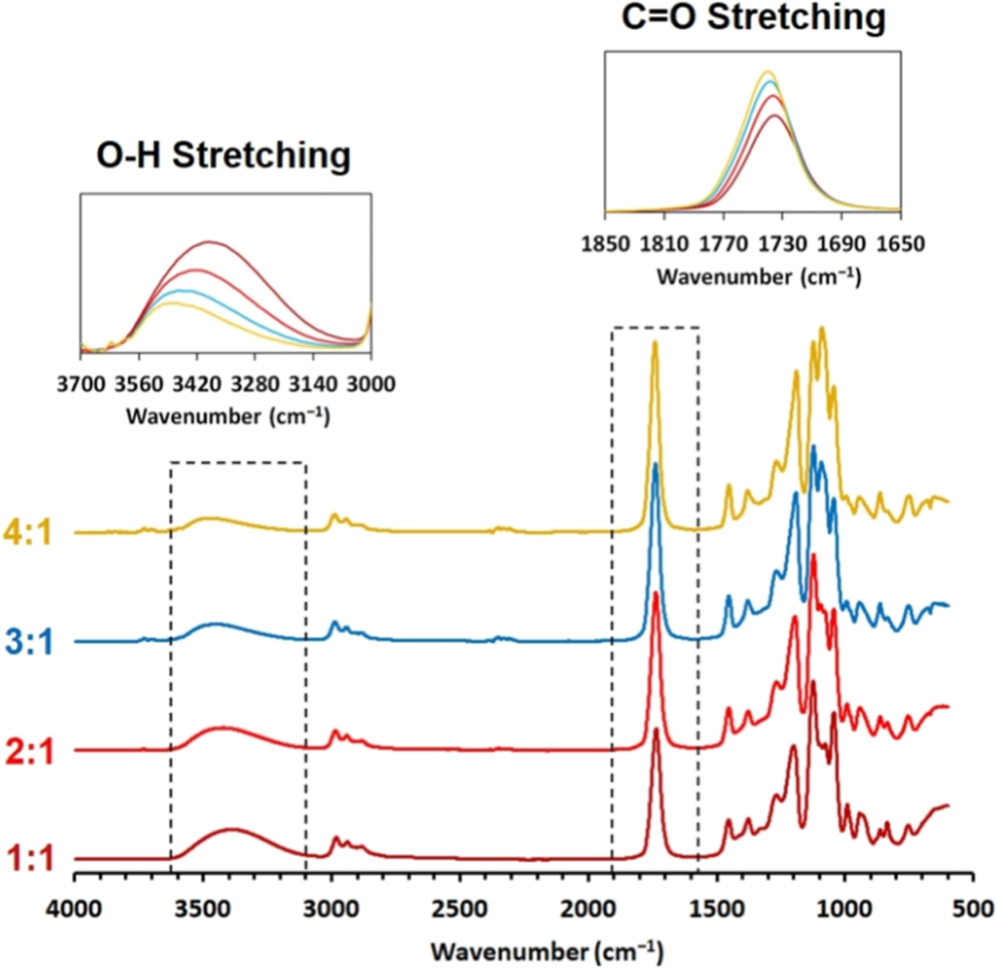
FTIR spectrum comparison of each PLA Oligo synthesized batch containing various reactant molar contents. The molar ratios in the graphs are PLA/PG.

After verification of the transesterified and methacrylated PLA structure, the one-pot syntheses of PLA Oligo followed by PLA MMA were performed. The catalytic transesterification of scrap PLA using PG, resulting in curable structure production, has not yet been introduced. Specific references using PLA oligomers with different hydroxyl functionalities were synthesized from a virgin material without implementing an upcycling step.^26^ The efficiency, reactants’ availability, and water solubility of the used catalyst titanium(IV) butoxide favor our proposed depolymerization route compared to the published PLA aminolysis.^20^ Also, PG as a reactant is purchasable in biobased grade, which can increase the biobased content in produced mixtures. The PLA Oligo molecular length differences summarize the hydroxyl values, shown in Table 1. The structural differences are projected by FTIR spectral comparison in Figure 3.

The PLA Oligo FTIR spectrum comparison confirms that the hydroxyl groups decrease with the increasing PLA oligomer content (4:1 ratio) due to the higher molecular weight (evident from the hydroxyl value in Table 1) with the same functionality of two hydroxyl groups per molecule (confirmed by cross-analysis). The hydroxyl value decreases with the increasing PLA ratio, verifying the volumetry for all PLA Oligo systems. Additionally, Figure 3 represents the increasing C=O stretching ester signal correlating with the rising PLA content in the mixture. This result confirms the increasing ester character per molecule due to a higher PLA content as a linear polyester structure. The results summarized the increasing molecular length with increasing PLA content in mixtures.

**Figure 4:**
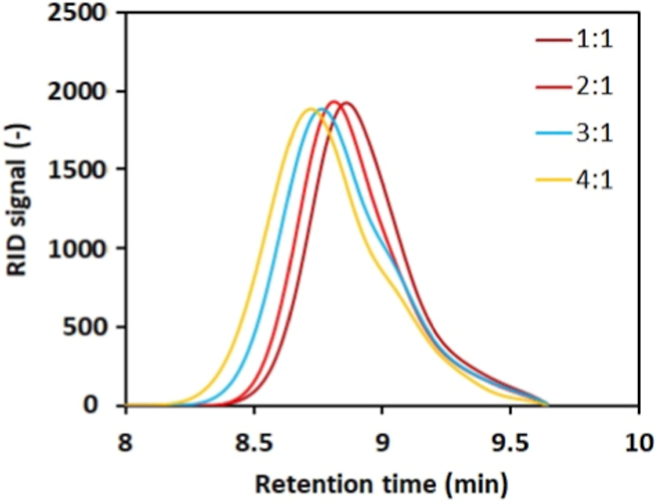
Chromatograms of PLA MMA-synthesized structures. The obtained values of number (*M*_n_) and weight (*M*_w_), average molecular weight, and PDI are shown in Table 1.

The structure length represented by weight (*M*_w_)- and number (*M*_n_)-average molecular weight was investigated via GPC. Due to the inappropriate solubility and polar character of PLA Oligo, GPC analysis was applied to the eventual PLA MMA curable structures. The hydroxyl values provide information regarding the PLA Oligo molecular length. The GPC chromatograms of each PLA MMA structure are summarized in Figure 4, and the results of the GPC analysis are shown in Table 1.

**Figure 5:**
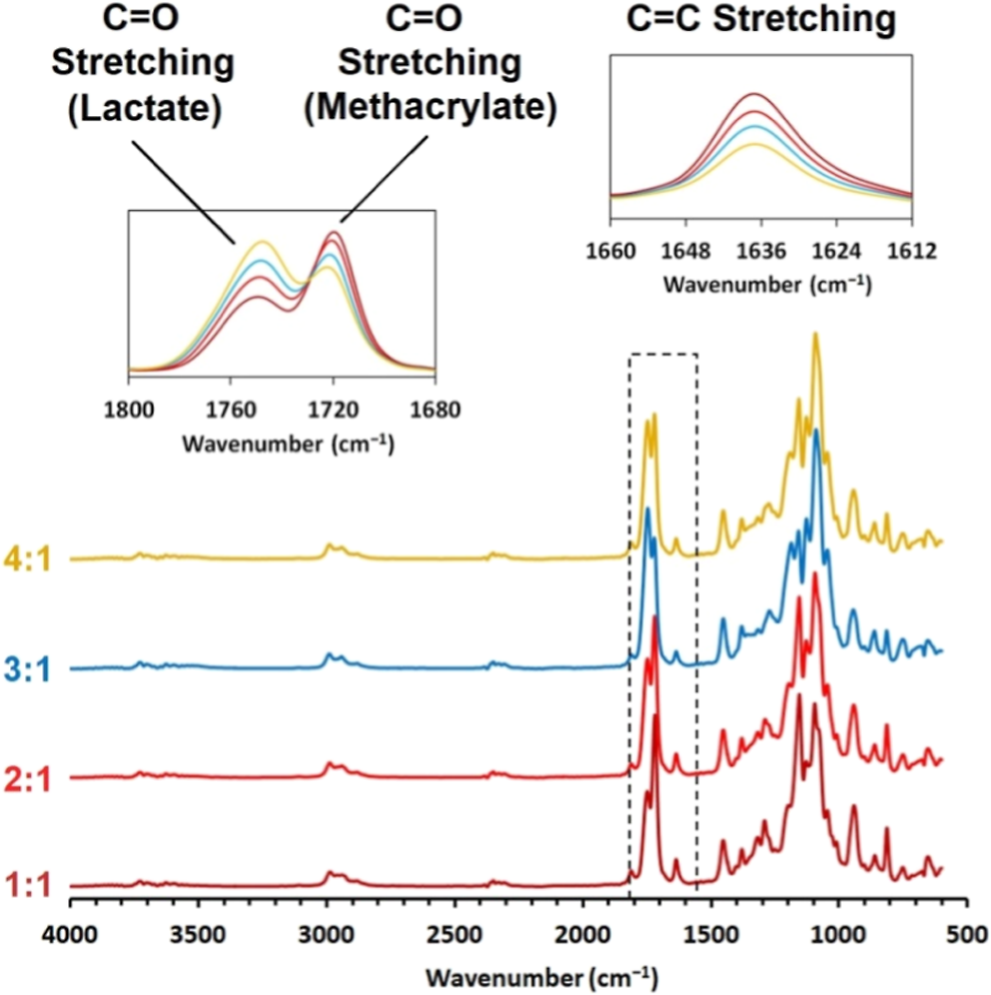
FTIR spectrum comparison of each PLA MMA-synthesized batch containing various reactant molar contents. The molar ratios in the graphs are PLA/PG.

The GPC analysis confirmed the increasing PLA MMA molecular length with an increasing PLA content in the depolymerization mixture. The structural character of produced PLA MMA systems was investigated using the FTIR method, similar to that of PLA Oligo systems. The GPC analysis can analyze the increasing molecular length, and the FTIR method uncovers the presence of curable double bonds located in the methacrylate functional groups. The results of the PLA MMA FTIR spectrum comparison are summarized in Figure 5.

The FTIR spectrum comparison provided complex information about PLA MMA structure changes. The hydroxyl O–H stretching signal (evident in Figure 3 at 3550–3200 cm^–1^ wavelength) is absent in PLA MMA FTIR results. This outcome confirms the complete conversion of the hydroxyl functional groups. Also, C=C stretching wavenumber interval 1662–1626 cm^–1^ verifies the methacrylate functional groups in PLA MMA structure. The comparison also displays the decrease of the C=C stretching signal with the increase in the PLA molar content. This information confirms the lesser molar methacrylate content in structures with higher molecular weight.^27^ Regarding the C=O stretching signal (1750–1720 cm^–1^) signalizing the ester bonding, the signal increases with the PLA molar ratio for an absorption maximum of 1750 cm^–1^, while it decreases for the second maximum at 1720 cm^–1^. The increase at 1750 cm^–1^ refers to the rise of lactate ester bonding, while the 1720 cm^–1^ maximum decrease refers to the methacrylate ester bonding, which is structurally less dominant due to the decreasing molar presence of methacrylate groups with increasing PLA content.^28^ The structural cross-analysis of PLA Oligo and PLA MMA monomers in connection with the GPC and FTIR PLA Oligo and PLA MMA comparisons summarized the complex report regarding the chemical production of curable upcycled scrap PLA.

### 3.2 Rheological Characterization of Synthesized Systems

**Figure 6:**
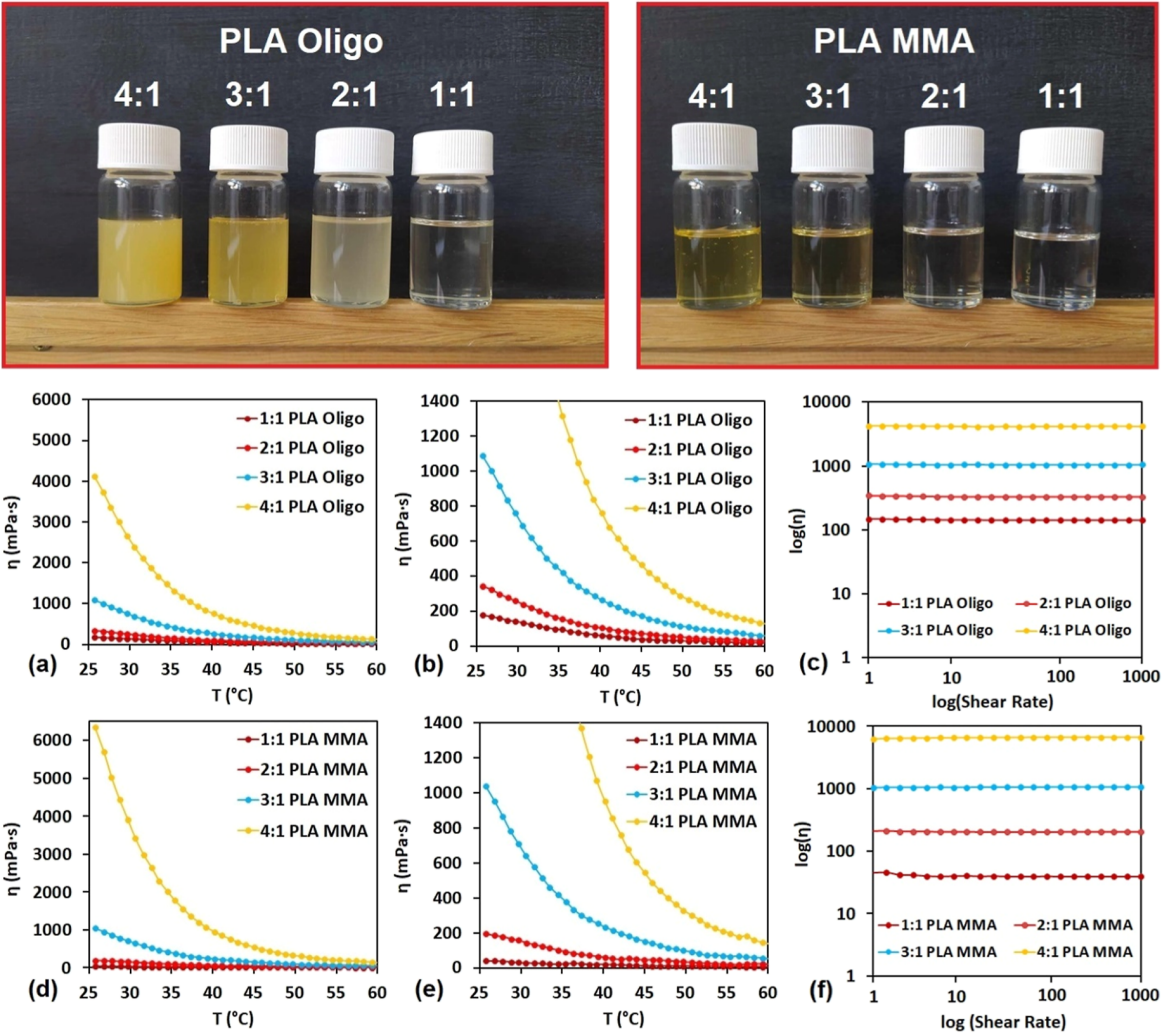
Rheological measurements of all produced systems. (a) PLA Oligo apparent viscosity dependency on temperature up to 6000 mPa·s; (b) detailed PLA Oligo apparent viscosity dependency on temperature up to 1400 mPa·s; (c) PLA Oligo apparent viscosity dependency on shear rate; (d) PLA MMA apparent viscosity dependency on temperature up to 6000 mPa·s; (e) detailed PLA MMA apparent viscosity dependency on temperature up to 1400 mPa·s; and (f) PLA MMA apparent viscosity dependency on shear rate.

The rheological behavior profile is crucial for every chemical process, including the synthesis efficiency and evaluation of the product’s potential applicability. The viscosity levels of PLA Oligo affect the production process and the limitations regarding the reactant or catalyst homogenization, while PLA MMA rheology directly determines the usage for SLA 3D printing. Specific functional groups’ functionality, molecular length, or presence predisposes the system’s rheological profile.^26^ PLA Oligo structures possess lower molecular length than PLA MMA, while the hydroxyl group functionality is present in PLA Oligo structures, in contrast to PLA MMA, where polar hydroxyl groups forming H-bonding are absent. The rheological study aims to describe the produced mixtures’ working characteristics. The dependency of apparent viscosity on the rising temperature and shear rate was investigated. The temperature influence is essential for the production process and the SLA 3D printing purposes, while the viscosity relation to changing shear rate can uncover the potential non-Newtonial behavior, which is typical for the polymer and oligomer solutions and melts.^29^ The results of PLA Oligo and PLA MMA rheological characterization are shown in Figure 6.

The rheological study uncovered changes in flow properties with varying product molecular structures. The polar water-soluble PLA Oligo exhibited in the 1:1 system an apparent viscosity value of 142 mPa·s at 30 °C, while the 1:1 PLA MMA system reached 31 mPa·s at the same temperature. This trend of higher PLA Oligo viscosity compared to PLA MMA is similar for the 2:1 systems (a 2:1 PLA Oligo value of 259 mPa·s and a 2:1 PLA MAA value of 158 mPa·s). The apparent viscosity levels reached practically identical values for 3:1 systems (PLA Oligo 761 mPa·s and PLA MMA 711 mPa·s). Eventually, 4:1 systems changed the characteristics, where 4:1 PLA Oligo exhibited an apparent viscosity of 2666 mPa·s at 30 °C, while the 4:1 PLA MMA mixture displayed a 3911 mPa·s value. The results confirm the increasing molecular length role connected to the flow properties. The short-chain polar PLA Oligo molecules are primarily forming more molecular interactions due to the H-bonding of hydroxyl groups.^30^ On the other hand, the long-chain 4:1 molecule rheological profile is mainly enhanced by the van der Waals additive interactions, which increase with the molecular size.^31^ The apparent viscosity dependency on the shear rate uncovered that all PLA Oligo and PLA MMA systems exhibit constant viscosity with varying shear rates. This behavior is typical for short-chain systems contrary to primarily pseudoplastic behavior of log chain polymer melts or solutions.^32^ The rheological study results, including the calculated Arrhenius parameters, are listed in Table 3.

**Table 3:** PLA Oligo and PLA MMA Rheological Characterization Results Including the Apparent Viscosity Values at 30 °C, Pre-exponential Factor Values, and the Flow Activation Energy Levels

Arrhenius function parameters				
system	η_30°C_ (mPa·s)	η_∞_ (mPa·s)	*E*_η_ (kJ/(mol·K))	*R*^2^
1:1 PLA Oligo	142	4.94 × 10^–11^	54.8	0.996
2:1 PLA Oligo	259	1.44 × 10^–11^	59.2	0.996
3:1 PLA Oligo	761	5.64 × 10^–13^	70.1	0.994
4:1 PLA Oligo	2666	9.36 × 10^–15^	83.6	0.982
1:1 PLA MMA	31	3.02 × 10^–8^	46.7	0.994
2:1 PLA MMA	158	6.93 × 10^–11^	53.9	0.993
3:1 PLA MMA	711	1.95 × 10^–13^	72.6	0.974
4:1 PLA MMA	3911	6.00 × 10^–16^	91.4	0.971

The results in Table 3 are calculated from the linearized forms of rheological investigations (the graphical interpretation of Arrhenius plots is provided in Figures S7 and S8). The pre-exponential factor decreases with rising apparent viscosity at 30 °C, while the flow activation energy increases since this parameter represents the system’s flow efficiency and reaches higher values for high-viscous systems.^23^ The flow activation energy describes the energetic barrier that the system needs to overcome to initiate its flow.^33^

### 3.3 Thermomechanical Properties of PLA MMA RESIN Systems

**Figure 7:**
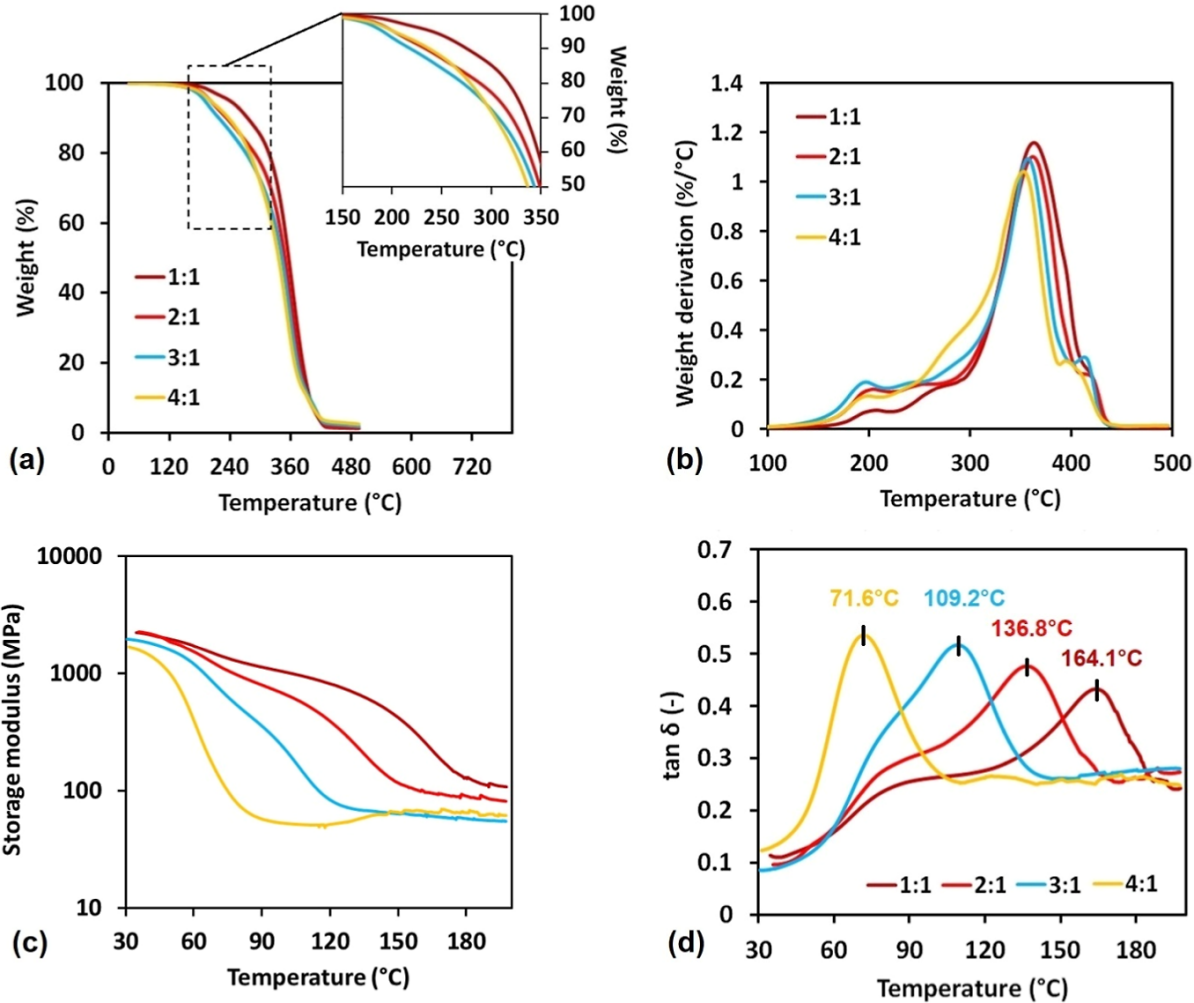
PLA MMA RESIN’s thermomechanical characterization. (a) TGA integral form with detailed segment at 150–350 °C temperature interval; (b) TGA derivative interpretation; (c) measured PLA MMA RESIN’s storage modules dependency on temperature; and (d) determined PLA MMA RESIN’s glass-transition temperatures from tan δ.

The thermomechanical property study provides information regarding the produced curable PLA MMA system performance and thermal stability. PLA MMA RESIN’s storage modules and glass-transition temperatures obtained from DMA represent the mechanical properties of each cured material. The calculated cross-linking densities refer to the particular cured system’s molecular site character. TGAs provided the PLA MMA RESIN’s thermal stability profile and served as the data for the heat-resistant index calculation. The graphical results of DMA and TGA are shown in Figure 7, and the measured and calculated parameters are summarized in Table 4. The PLA content and the renewable content were calculated according to the initial reaction mixture’s composition and the expected methacrylated compounds’ molecular weight ratio to the measured and investigated compounds after the transesterification. PLA and PG are considered to be renewable sources based on their origin in production. The additional thermomechanical characterization results are shown in Figure S9.

**Table 4:** Thermomechanical Characterization Results Including Measured and Calculated Parameters from DMA and TGA

system molar ratio (PLA/PG)	DMA			TGA				composition	
	*E*_30°C_^′^ (MPa)	*T*_g_ (°C)	ν_e_ (kmol/m^3^)	*T*_5_ (°C)	*T*_30_ (°C)	*T*_max_ (°C)	*T*_S_ (°C)	PLA content (wt %)	renewable content (wt %)
1:1	2240	164.1	9.06	239.7	335.4	363.1	145.6	26.1	53.3
2:1	2270	136.8	7.97	201.3	320.7	361.3	133.7	41.6	63.2
3:1	1960	109.2	6.05	190.3	307.1	357.0	127.6	51.3	69.0
4:1	1680	71.6	5.21	201.5	303.9	352.8	128.8	61.0	76.8

The DMA results uncover the mechanical performance decreasing trend with the rising PLA content in the system. Systems 1:1 and 2:1 reached practically identical storage modulus values, and following systems, *E*′ values decreased by 310 and 570 MPa for 3:1 and 4:1, respectively. The decreasing glass-transition temperature follows the same trend as the storage modulus, except that the decrease is also evident between the 1:1 and 2:1 systems. The cross-linking densities calculated from DMA measured data show a total reduction of 42.5% in the case of a 4:1 system compared to that of 1:1. All these results confirm the rising molecular mobility character of long-chain cured systems (mainly 4:1), leading to the less rigid and cross-linked molecular cured site.^23^ The results verify that measured values obtained from DMA are competitive with the commercial SLA resins such as ANYCUBIC (the *E*_30°C_^′^ value reaches 1700–1500 MPa, and *T*_g_ is 79.2 °C) or MONOPRICE (the *E*_30°C_^′^ value reaches 1500–1300 MPa, and *T*_g_ is 88.9 °C).^20^ Additionally, PLA MMA RESINs do not require reactive diluents, contrary to aminolyzed PLA in published experiments.^20^

PLA MMA RESIN’s thermal stability reached lower values in comparison with ANYCUBIC and MONOPRICE. The comparable *T*_5_ values reached 239.1 °C for the most stable 1:1 system and 190.3 °C for 3:1 PLA MMA RESIN. The mentioned commercial SLA resins reached the approximal *T*_5_ value of 325 °C.^20^ The decreasing trend in the PLA thermal stability is directly connected to the PLA content within the cured molecular structure since polyester bonding is not optimally stable in high-temperature working conditions.^34^ However, since SLA 3D printing is usually performed at room temperature, the lesser PLA MMA RESIN heat resistance does not limit the upcycled resin’s potential for various SLA-printed products in numerous application fields. Additionally, biobased ester systems often tend to exhibit lower thermal stability.^35^

### 3.4 SLA Printing of Produced PLA MMA Systems

**Figure 8:**
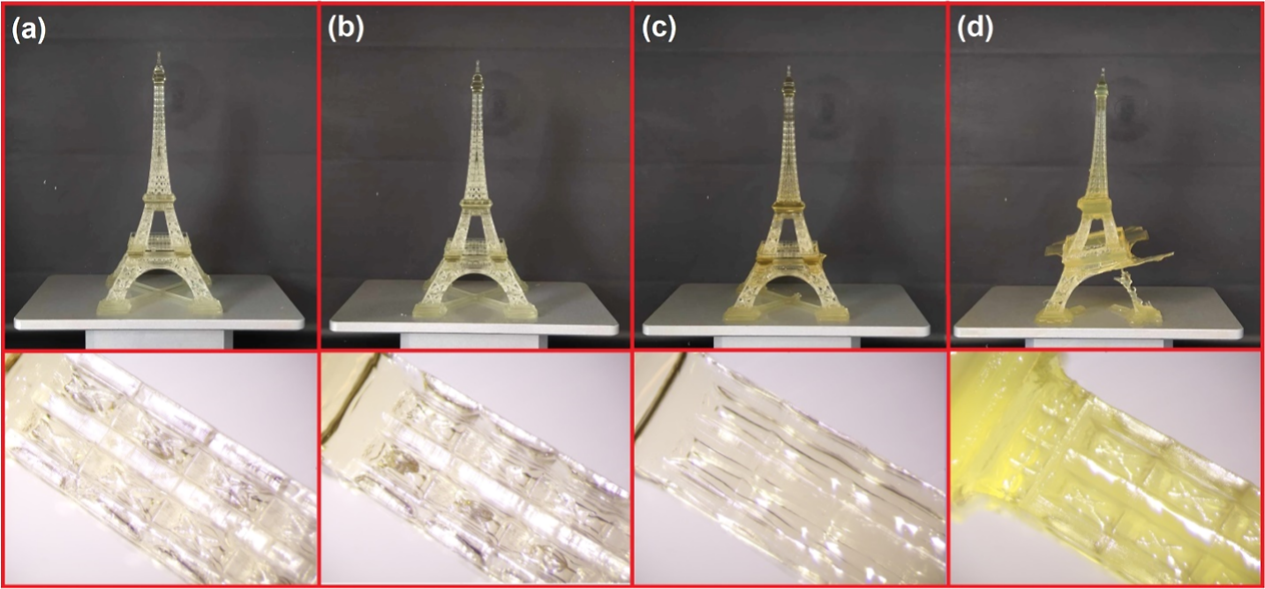
Printed prototypes from synthesized curable systems using stereolithography (SLA). (a) PLA MMA 1:1; (b) PLA MMA 2:1; (c) PLA MMA 3:1; and (d) PLA MMA 4:1.

PLA MMA printability is the system’s fundamental property since its applicability was targeted toward stereolithography. When DMA and TGA testing specimens were prepared, the system’s curability was confirmed. However, sufficient reactivity, adhesion, and decent viscosity levels determine whether the resin is suitable for SLA purposes.^36^ The printing was experimentally tested with PLA MMA systems containing 1 wt % BAPO due to its lesser environmental impact. Commonly, BAPO content in SLA resins reaches up to 4.5 wt %.^36^ The printed prototypes are shown in Figure 8. The printed details rapidly decrease with the PLA content of the resin. The 1:1 PLA MMA system (Figure 8a) showed extremely high resolution and proved significantly appropriate for SLA. The SLA applicability was confirmed for systems 2:1 and 3:1 PLA MMA (Figure 8b,c, respectively) since these systems created the whole printed prototypes. The system 4:1 PLA MMA (Figure 8d) is not suitable for SLA since the printed object collapsed during the process. The high viscosity of the system primarily caused this outcome. The reactivity was not the critical factor since the object was eventually printed, but it leaned to the side and did not possess sufficient resolution. The SLA printability test uncovered that PLA MMA 1:1, 2:1, and 3:1 can serve as an SLA resin, while PLA MMA 4:1 is not optimal for this purpose.

**Figure 9:**
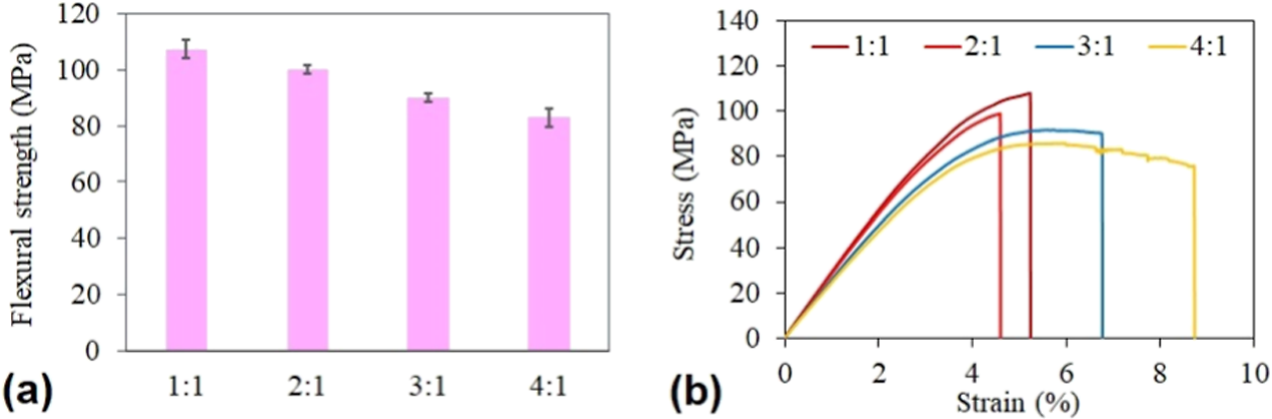
Flexural test of printed PLA MMA RESINs. (a) Summarized values of flexural strength including the error bars and (b) selected stress–strain curves for all measured systems.

We performed additional mechanical flexural tests on the 3D printed specimens to observe the prepared resins’ functional properties. The results are shown in Figure 9. We performed six measurements for every cured system according to the norm. The results reached in Figure 9a uncover the same mechanical trend as the DMA results. The 1:1 PLA MMA RESIN exhibited the highest flexural strength, reaching 107 ± 3 MPa, while the lowest value was measured for 4:1 PLA MMA RESIN, reaching 83 ± 3 MPa. While the mechanical property trend was maintained, the performance of all prepared PLA MMA RESINs is significantly better compared to the reported ANYCUBIC and MONOPRICE resins. The authors in the cited publication^20^ observed the engineering stress of ANYCUBIC cured resin reaching 22 MPa and MONOPRICE’s value of 48 MPa. Also, the mechanical properties’ investigation was not affected by a significant degree of cure deviations since all PLA MMA RESINs reached approximately 90% degree of cure (the lowest value 1:1 PLA MMA RESIN was 89.4%, and the biggest value 4:1 PLA MMA RESIN was 91.4%). The flexural test confirmed the trends observed in the previously performed analyses.

## 4 Conclusions

This work provided a potential upcycling route for waste PLA material obtained from FDM 3D printing filaments. The transesterified PLA oligomers were obtained by PLA depolymerization by using PG as a reactant. The PLA oligomers were depolymerized in different molar ratios, forming variably long molecules analyzed by GPC. The produced PLA oligomers were modified by methacrylic anhydride to form viscous systems capable of being photoinitially cured. All produced systems were cross-analyzed by using NMR, ESI-MS, and FTIR analyses. The rheological characterization uncovered the rapid viscosity changes connected to the molecular lengths and systems’ polarity. The lowest viscous curable system reached 31 mPa·s at 30 °C, while the highest value was 3911 mPa·s at 30 °C. All synthesized curable systems reached sufficient thermomechanical properties comparable to the commercial SLA resins, ANYCUBIC, and MONOPRICE. The storage modules reached 1680–2270 MPa, and the measured glass-transition temperatures were 71.6–164.1 °C. The best flexural strength exhibited PLA MMA 1:1, reaching 107 ± 3 MPa; the lowest value was measured for PLA MMA 4:1, reaching 83 ± 3 MPa. The produced curable systems were used as SLA printing resins. The PLA MMA 4:1 system containing the highest waste PLA content (61.0 wt %) is unsuitable for SLA 3D printing due to the high viscosity levels. Except for this system, all systems were successfully used for SLA and turned out to be promising resins containing upcycled PLA content.

## Supporting Information Available

Additional data regarding structural verification, graphical interpretation of the Arrhenius plot, DMA data, and flexural test data (PDF)

## References

[ref1] Yu J.; Xu S.; Liu B.; Wang H.; Qiao F.; Ren X.; Wei Q. Pla Bioplastic Production: From Monomer To The Polymer. Eur. Polym. J. 2023, 193, 112076 10.1016/j.eurpolymj.2023.112076.

[ref2] DeStefano V.; Khan S.; Tabada A. Applications Of Pla In Modern Medicine. Eng. Regen. 2020, 1, 76–87. 10.1016/j.engreg.2020.08.002. 38620328 PMC7474829

[ref3] Ebrahimi F.; Ramezani Dana H. Poly Lactic Acid (Pla) Polymers: From Properties To Biomedical Applications. Int. J. Polym. Mater. Polym. Biomater. 2022, 71 (15), 1117–1130. 10.1080/00914037.2021.1944140.

[ref4] Yang Y.; Zhang M.; Ju Z.; Tam P. Y.; Hua T.; Younas M. W.; Kamrul H.; Hu H. Poly(Lactic Acid) Fibers, Yarns And Fabrics: Manufacturing, Properties And Applications. Text. Res. J. 2021, 91 (13–14), 1641–1669. 10.1177/0040517520984101.

[ref5] Bhagia S.; Bornani K.; Agrawal R.; Satlewal A.; Ďurkovič J.; Lagaňa R.; Bhagia M.; Yoo C. G.; Zhao X.; Kunc V.; Pu Y.; Ozcan S.; Ragauskas A. J. Critical Review Of Fdm 3D Printing Of Pla Biocomposites Filled With Biomass Resources, Characterization, Biodegradability, Upcycling And Opportunities For Biorefineries. Appl. Mater. Today 2021, 24, 101078 10.1016/j.apmt.2021.101078.

[ref6] Cvek M.; Paul U. C.; Zia J.; Mancini G.; Sedlarik V.; Athanassiou A. Biodegradable Films Of Pla/Ppc And Curcumin As Packaging Materials And Smart Indicators Of Food Spoilage. ACS Appl. Mater. Interfaces 2022, 14 (12), 14654–14667. 10.1021/acsami.2c02181. 35302368 PMC8972250

[ref7] da Costa J. P.; Mouneyrac C.; Costa M.; Duarte A. C.; Rocha-Santos T. The Role Of Legislation, Regulatory Initiatives And Guidelines On The Control Of Plastic Pollution. Front. Environ. Sci. 2020, 8, 104 10.3389/fenvs.2020.00104.

[ref8] Valvez S.; Santos P.; Parente J. M.; Silva M. P.; Reis P. N. B. 3D Printed Continuous Carbon Fiber Reinforced Pla Composites: A Short Review. Procedia Struct. Integr. 2020, 25, 394–399. 10.1016/j.prostr.2020.04.056.

[ref9] Ahuja R.; Kumari N.; Srivastava A.; Bhati P.; Vashisth P.; Yadav P. K.; Jacob T.; Narang R.; Bhatnagar N. Biocompatibility Analysis Of Pla Based Candidate Materials For Cardiovascular Stents In A Rat Subcutaneous Implant Model. Acta Histochem. 2020, 122 (7), 151615 10.1016/j.acthis.2020.151615. 33066837

[ref10] Liu S.; Qin S.; He M.; Zhou D.; Qin Q.; Wang H. Current Applications Of Poly(Lactic Acid) Composites In Tissue Engineering And Drug Delivery. Composites, Part B 2020, 199, 108238 10.1016/j.compositesb.2020.108238.

[ref11] DeStefano V.; Khan S.; Tabada A. Applications Of Pla In Modern Medicine. Eng. Regen. 2020, 1, 76–87. 10.1016/j.engreg.2020.08.002. 38620328 PMC7474829

[ref12] Rahmatabadi D.; Ghasemi I.; Baniassadi M.; Abrinia K.; Baghani M. 3D Printing Of Pla-Tpu With Different Component Ratios: Fracture Toughness, Mechanical Properties, And Morphology. J. Mater. Res. Technol. 2022, 21, 3970–3981. 10.1016/j.jmrt.2022.11.024.

[ref13] Afonso J. A.; Alves J. L.; Caldas G.; Gouveia B. P.; Santana L.; Belinha J. Influence Of 3D Printing Process Parameters On The Mechanical Properties And Mass Of Pla Parts And Predictive Models. Rapid Prototyp. J. 2021, 27 (3), 487–495. 10.1108/RPJ-03-2020-0043.

[ref14] Chen X.; Wang Y.; Zhang L. Recent Progress In The Chemical Upcycling Of Plastic Wastes. ChemSusChem 2021, 14 (19), 4137–4151. 10.1002/cssc.202100868. 34003585

[ref15] Chen H.; Wan K.; Zhang Y.; Wang Y. Waste To Wealth: Chemical Recycling And Chemical Upcycling Of Waste Plastics For A Great Future. ChemSusChem 2021, 14 (19), 4123–4136. 10.1002/cssc.202100652. 33998153

[ref16] Storozhuk I. P.; Pavlukovich N. G.; Polezhaev A. V. Polytetrahydrofuran From Renewable Natural Raw Materials And Polymers Based On It. J. Phys.: Conf. Ser. 2021, 1990 (1), 012034 10.1088/1742-6596/1990/1/012034.

[ref17] Zhang X.; Sun Y.; Zhang C.; Zhang X. Upcycling Polytetrahydrofuran To Polyester. CCS Chem. 2023, 5 (5), 1233–1241. 10.31635/ccschem.022.202202072.

[ref18] Coates G. W.; Getzler Y. D. Y. L. Chemical Recycling To Monomer For An Ideal, Circular Polymer Economy. Nat. Rev. Mater. 2020, 5 (7), 501–516. 10.1038/s41578-020-0190-4.

[ref19] Ji L.; Meng J.; Li C.; Wang M.; Jiang X. From Polyester Plastics To Diverse Monomers Via Low-Energy Upcycling. Adv. Sci. 2024, 11, 2403002 10.1002/advs.202403002. PMC11220695 38626364

[ref20] Shao L.; ChangHao Y. C.; Fei M. e.; ZhaoBlissZhang B. B. J. J.; Zhao B.; Bliss B. J.; Zhang J. A. A chemical approach for the future of PLA upcycling: from plastic wastes to new 3D printing materials. Green Chem. 2022, 24 (22), 8716–8724. 10.1039/D2GC01745H.

[ref21] Wang F.; Allen D.; Tian S.; Oler E.; Gautam V.; Greiner R.; Metz T. O.; Wishart D. S. Cfm-Id 4.0 – A Web Server For Accurate Ms-Based Metabolite Identification. Nucleic Acids Res. 2022, 50 (W1), W165–W174. 10.1093/nar/gkac383. 35610037 PMC9252813

[ref22] Jašek V.; Fučík J.; Melcova V.; Figalla S.; Mravcova L.; Krobot S. ˇ.; Přikryl R. Synthesis of Bio-Based Thermoset Mixture Composed of Methacrylated Rapeseed Oil and Methacrylated Methyl Lactate: One-Pot Synthesis Using Formed Methacrylic Acid as a Continual Reactant. Polymers 2023, 15, 1811 10.3390/polym15081811. 37111957 PMC10146403

[ref23] Liu W.; Xie T.; Qiu R. Biobased Thermosets Prepared From Rigid Isosorbide And Flexible Soybean Oil Derivatives. ACS Sustainable Chem. Eng. 2017, 5 (1), 774–783. 10.1021/acssuschemeng.6b02117.

[ref24] Ibrahim E.; Ahmed S.; Abir S. S. H.; Taylor K.; Padilla-Gainza V. M.; Lozano K. Centrifugally Spun Alginate-Poly(Lactic Acid) Microbeads: A Promising Carrier For Drug Delivery And Tissue Engineering. Int. J. Biol. Macromol. 2022, 220, 671–682. 10.1016/j.ijbiomac.2022.08.097. 35988730

[ref25] Zhang C.; Madbouly S. A.; Kessler M. R. Biobased Polyurethanes Prepared From Different Vegetable Oils. ACS Appl. Mater. Interfaces 2015, 7 (2), 1226–1233. 10.1021/am5071333. 25541678

[ref26] Esmaeili N.; Jahandideh A.; Muthukumarappan K.; Åkesson D.; Skrifvars M. Synthesis And Characterization Of Methacrylated Star-Shaped Poly(Lactic Acid) Employing Core Molecules With Different Hydroxyl Groups. J. Appl. Polym. Sci. 2017, 134 (39), 45341 10.1002/app.45341.

[ref27] Delgado A. H. S.; Young A. M.; Deshpande P. A. Methacrylate Peak Determination And Selection Recommendations Using Atr-Ftir To Investigate Polymerisation Of Dental Methacrylate Mixtures. PLoS One 2021, 16 (6), e0252999 10.1371/journal.pone.0252999. 34106972 PMC8189511

[ref28] Lee B.-S.; Chen Y.-J.; Wei T.-C.; Ma T.-L.; Chang C.-C. Comparison Of Antibacterial Adhesion When Salivary Pellicle Is Coated On Both Poly(2-Hydroxyethyl-Methacrylate)- And Polyethylene-Glycol-Methacrylate-Grafted Poly(Methyl Methacrylate). Int. J. Mol. Sci. 2018, 19 (9), 2764 10.3390/ijms19092764. 30223440 PMC6164387

[ref29] Nofar M.; Salehiyan R.; Sinha Ray S. Rheology Of Poly (Lactic Acid)-Based Systems. Polym. Rev. 2019, 59 (3), 465–509. 10.1080/15583724.2019.1572185.

[ref30] Wang D.; Wang R.; Chen S.; Gao J.; Cai C.; Zheng Y.; Liu X.; Qu B.; Chen N.; Zhuo D. Low Viscosity And Highly Flexible Stereolithographic 3D Printing Resins For Flexible Sensors. Mater. Des. 2024, 243, 113052 10.1016/j.matdes.2024.113052.

[ref31] Huang X.; Peng S.; Zheng L.; Zhuo D.; Wu L.; Weng Z. 3D Printing Of High Viscosity Uv-Curable Resin For Highly Stretchable And Resilient Elastomer. Adv. Mater. 2023, 35 (49), 2304430 10.1002/adma.202304430. 37527974

[ref32] Naser A. Z.; Deiab I.; Darras B. M. Poly(Lactic Acid) (Pla) And Polyhydroxyalkanoates (Phas), Green Alternatives To Petroleum-Based Plastics: A Review. RSC Adv. 2021, 11 (28), 17151–17196. 10.1039/D1RA02390J. 35479695 PMC9033233

[ref33] Hamdi R.; Massoudi I.; Alotaibi D. H.; Ouerfelli N. Novel Linear/Nonlinear Dependence Between The Viscosity Arrhenius Parameters Correlation In Newtonian Liquids. Chem. Phys. 2021, 542, 111076 10.1016/j.chemphys.2020.111076.

[ref34] Santos J. M. C.; Marques D. S.; Alves P.; Correia T. R.; Correia I. J.; Baptista C. M. S. G.; Ferreira P. Synthesis, functionalization and characterization of UV-curable lactic acid based oligomers to be used as surgical adhesives. React. Funct. Polym. 2015, 94, 43–54. 10.1016/j.reactfunctpolym.2015.07.003.

[ref35] Barkane A.; Platnieks O.; Jurinovs M.; Gaidukovs S. Thermal Stability Of Uv-Cured Vegetable Oil Epoxidized Acrylate-Based Polymer System For 3D Printing Application. Polym. Degrad. Stab. 2020, 181, 109347 10.1016/j.polymdegradstab.2020.109347.

[ref36] Guerra A. J.; Lammel-Lindemann J.; Katko A.; Kleinfehn A.; Rodriguez C. A.; Catalani L. H.; Becker M. L.; Ciurana J.; Dean D. Optimization Of Photocrosslinkable Resin Components And 3D Printing Process Parameters. Acta Biomater. 2019, 97, 154–161. 10.1016/j.actbio.2019.07.045. 31352105

[ref37] Teixeira L. V.; Bomtempo J. V.; Oroski F. d. A.; Coutinho P. L. d. A. The Diffusion Of Bioplastics: What Can We Learn From Poly(Lactic Acid)?. Sustainability 2023, 15 (6), 4699 10.3390/su15064699.

[ref38] Jayasekara T.; Wickrama Surendra Y.; Rathnayake M. Polylactic Acid Pellets Production From Corn And Sugarcane Molasses: Process Simulation For Scaled-Up Processing And Comparative Life Cycle Analysis. J. Polym. Environ. 2022, 30 (11), 4590–4604. 10.1007/s10924-022-02535-w.

[ref39] Tan C.; Tao F.; Xu P. Direct Carbon Capture For The Production Of High-Performance Biodegradable Plastics By Cyanobacterial Cell Factories. Green Chem. 2022, 24 (11), 4470–4483. 10.1039/D1GC04188F.

[ref40] Hobbs S. R.; Parameswaran P.; Astmann B.; Devkota J. P.; Landis A. E. Anaerobic Codigestion Of Food Waste And Polylactic Acid: Effect Of Pretreatment On Methane Yield And Solid Reduction. Adv. Mater. Sci. Eng. 2019, 2019, 1–6. 10.1155/2019/4715904.

[ref41] Kwan T. H.; Hu Y.; Lin C. S. K. Techno-Economic Analysis Of A Food Waste Valorisation Process For Lactic Acid, Lactide And Poly(Lactic Acid) Production. J. Cleaner Prod. 2018, 181, 72–87. 10.1016/j.jclepro.2018.01.179.

[ref42] Swetha T. A.; Ananthi V.; Bora A.; Sengottuvelan N.; Ponnuchamy K.; Muthusamy G.; Arun A. A Review On Biodegradable Polylactic Acid (Pla) Production From Fermentative Food Waste - Its Applications And Degradation. Int. J. Biol. Macromol. 2023, 234, 123703 10.1016/j.ijbiomac.2023.123703. 36801291

[ref43] Rezvani Ghomi E. R.; Khosravi F.; Saedi Ardahaei A. S.; Dai Y.; Neisiany R. E.; Foroughi F.; Wu M.; Das O.; Ramakrishna S. The Life Cycle Assessment For Polylactic Acid (Pla) To Make It A Low-Carbon Material. Polymers 2021, 13 (11), 1854 10.3390/polym13111854. 34199643 PMC8199738

[ref44] Kalita N. K.; Damare N. A.; Hazarika D.; Bhagabati P.; Kalamdhad A.; Katiyar V. Biodegradation And Characterization Study Of Compostable Pla Bioplastic Containing Algae Biomass As Potential Degradation Accelerator. Environ. Challenges 2021, 3, 100067 10.1016/j.envc.2021.100067.

[ref45] Román-Ramírez L. A.; McKeown P.; Jones M. D.; Wood J. Kinetics Of Methyl Lactate Formation From The Transesterification Of Polylactic Acid Catalyzed By Zn(Ii) Complexes. ACS Omega 2020, 5 (10), 5556–5564. 10.1021/acsomega.0c00291. 32201849 PMC7081642

[ref46] Abdullah M. F.; Andriyana A.; Muhamad F.; Ang B. C. Fabrication Of Poly(Lactic Acid)-Cellulose Acetate Core-Shell Electrospun Fibers With Improved Tensile Strength And Biocompatibility For Bone Tissue Engineering. J. Polym. Res. 2023, 30 (7), 257 10.1007/s10965-023-03639-0.

[ref47] Abdullah M. F.; Andriyana A.; Muhamad F.; Ang B. C. Fabrication Of Poly(Lactic Acid)-Cellulose Acetate Core-Shell Electrospun Fibers With Improved Tensile Strength And Biocompatibility For Bone Tissue Engineering. J. Polym. Res. 2023, 30 (7), 257 10.1007/s10965-023-03639-0.

[ref48] Khouri N. G.; Bahú J. O.; Blanco-Llamero C.; Severino P.; Concha V. O. C.; Souto E. B. Polylactic Acid (Pla): Properties, Synthesis, And Biomedical Applications – A Review Of The Literature. J. Mol. Struct. 2024, 1309, 138243 10.1016/j.molstruc.2024.138243.

[ref49] Ramezani Dana H.; Ebrahimi F. Synthesis, Properties, And Applications Of Polylactic Acid-Based Polymers. Polym. Eng. Sci. 2023, 63 (1), 22–43. 10.1002/pen.26193.

[ref50] An introduction to bioplastics in medical applications. Medicinal Plastics News—Advanced Medical Plastics. 2023, https://www.medicalplasticsnews.com/medical-plastics-industry-insights/medical-plastics-materials-insights/an-introduction-to-bioplastics-in-medical-applications/.

