# Supplementary material for: Poly(lactide) Upcycling Approach through Transesterification for Stereolithography 3D Printing
Source: Biomacromolecules. 2024 Oct 3;25(10):6645–55. doi: 10.1021/acs.biomac.4c00840 (PMC11480983; doi:10.1021/acs.biomac.4c00840)
Supplement: Supplementary file 1 — bm4c00840_si_001.pdf [file bm4c00840_si_001.pdf]

Poly(lactide) (PLA) upcycling approach through transesterification for stereolithography 3D printing

Silvestr Figalla[a], Vojtěch Jašek[a*], Jan Fučík[b], Přemysl Menčík[a], Radek Přikryl[a]

a Institute of Materials Chemistry, Faculty of Chemistry, Brno University of Technology, 61200 Brno, Czech Republic.

b Institute of Environmental Chemistry, Faculty of Chemistry, Brno University of Technology, 612 00 Brno, Czech Republic

*corresponding author: xcjasekv@vutbr.cz

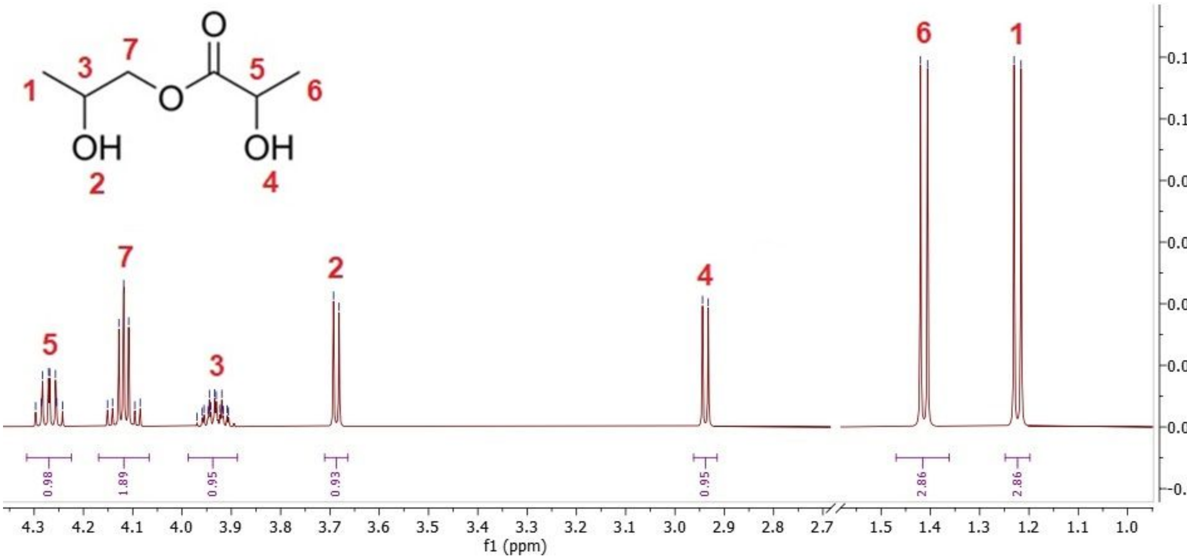

Figure S1. ^{1}H NMR (500 MHz, Chloroform-d) δ 4.27 (qd, J = 7.1, 5.9 Hz, 1H), 4.17 – 4.07 (m, 2H), 3.93 (dddd, J = 12.5, 6.9, 5.6, 1.7 Hz, 1H), 3.69 (d, J = 5.7 Hz, 1H), 2.94 (d, J = 5.9 Hz, 1H), 1.41 (d, J = 7.1 Hz, 3H), 1.22 (d, J = 7.1 Hz, 3H).

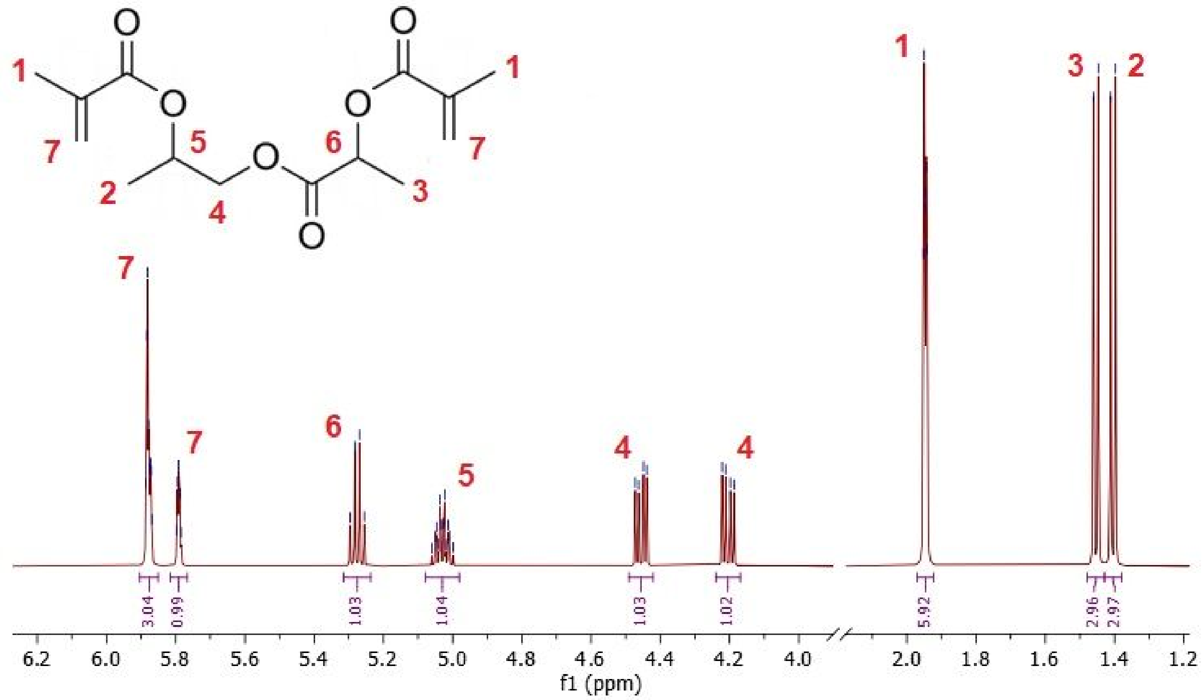

Figure S2. ^{1}H NMR of 2-hydroxypropyl 2-hydroxypropanoate methacrylate (500 MHz, Chloroform-d) δ 4.22 – 4.14 (m, 1H), 4.11 – 3.98 (m, 1H), 3.90 (ddd, J = 7.8, 6.4, 3.1 Hz, 1H), 3.61 (dd, J = 11.1, 3.1 Hz, 1H), 3.39 (dd, J = 11.1, 7.7 Hz, 1H), 1.48 – 1.36 (m, 3H), 1.30 – 1.19 (m, 3H).

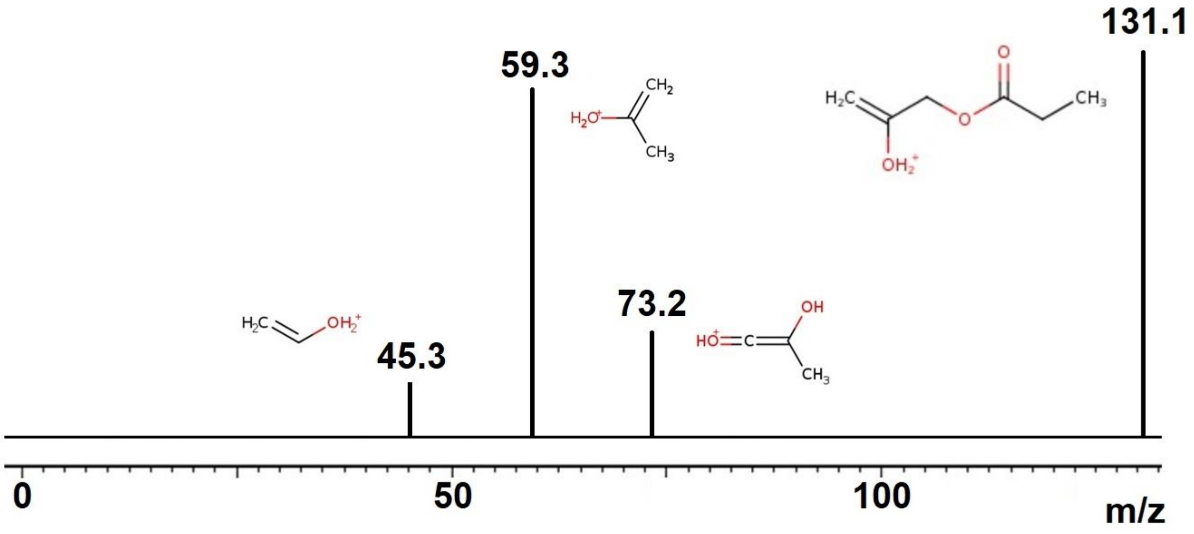

Figure S3. 2-hydroxypropyl lactate ESI-MS fragmentation spectrum ($C_6H_{12}O_4$) spectrum calc. [M − $H_2O]^+$ 131.07 m/z, found 131.10 m/z.

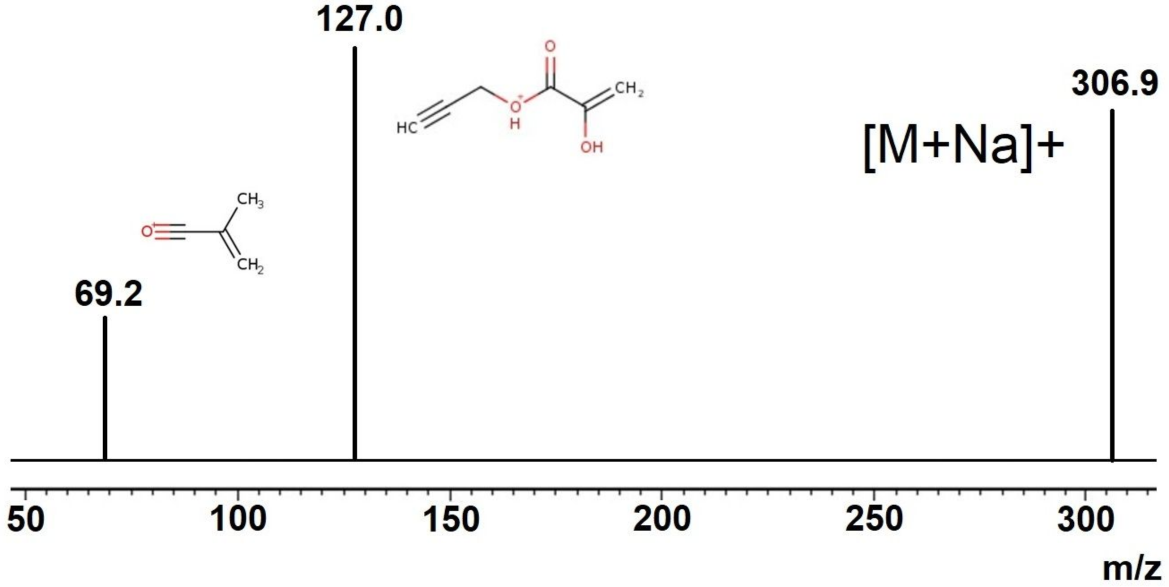

Figure S4. 2-hydroxypropyl lactate methacrylate ESI-MS fragmentation spectrum ($C_{14}H_{20}O_6Na$) spectrum calc. $[M + Na]^+$ 307.30 m/z, found 306.90 m/z.

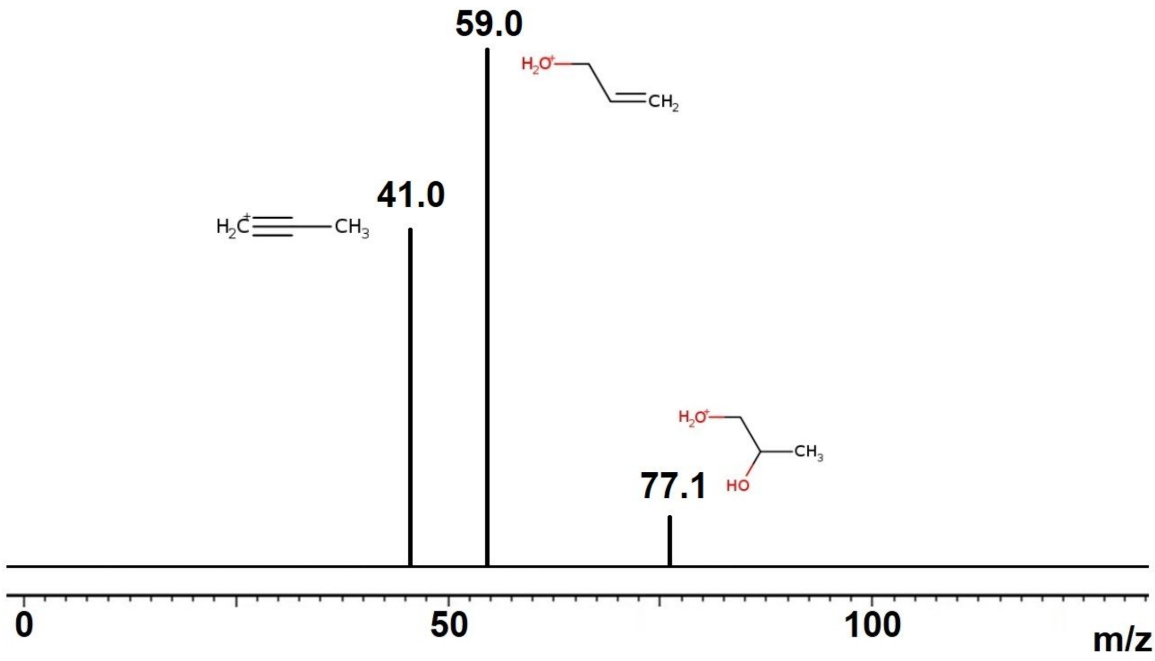

Figure S5. Propylene glycol ESI-MS fragmentation spectrum ($C_3H_8O_2$) spectrum calc. $[M + H]^+$ 77.06 m/z, found 77.10 m/z.

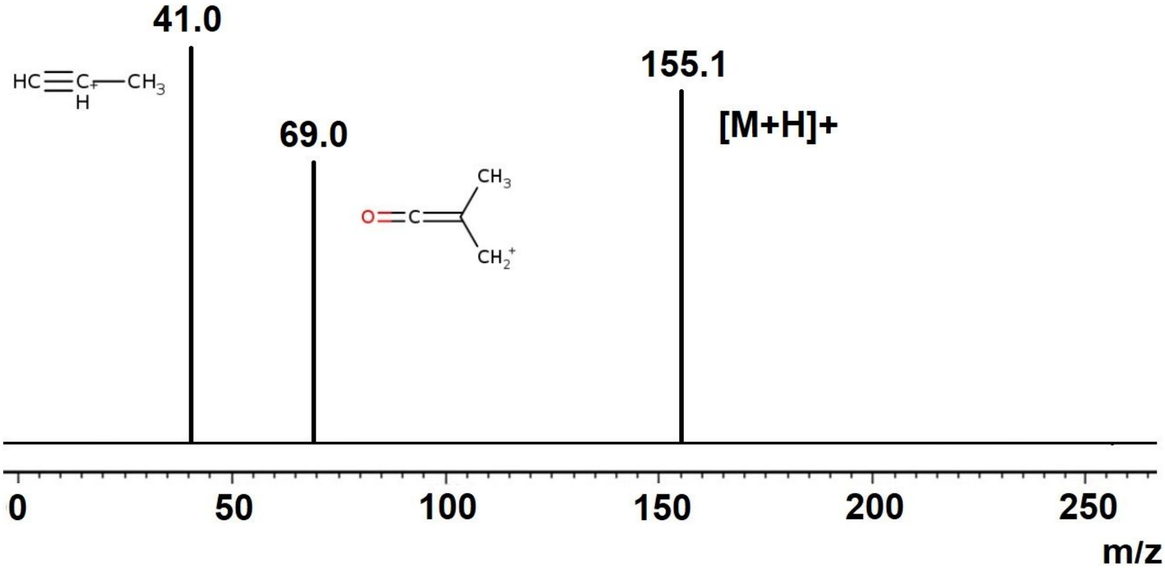

Figure S6. Methacrylic anhydride ESI-MS fragmentation spectrum ($C_8H_{10}O_3$) spectrum calc. $[M + H]^+$ 155.16 m/z, found 155.10 m/z.

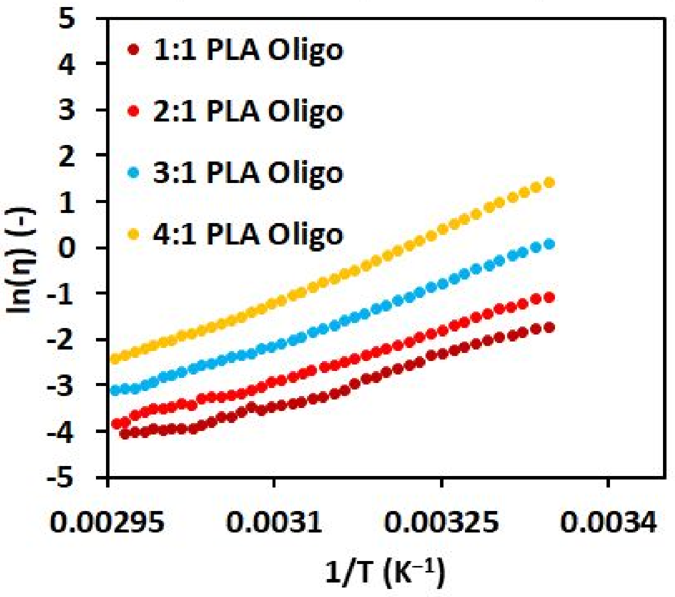

Figure S7. The graphical interpretation of PLA Oligo Arrhenius plot.

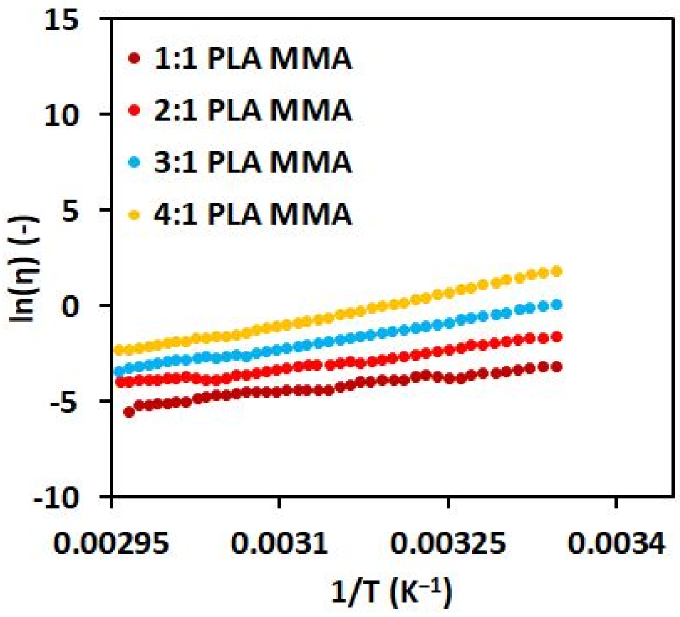

Figure S8. The graphical interpretation of PLA MMA Arrhenius plot.

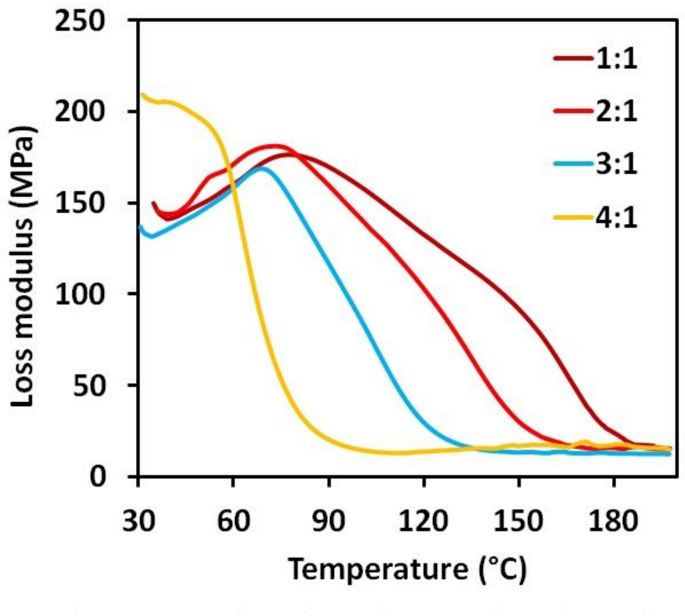

Figure S9. PLA MMA systems' loss modules obtained from DMA analysis.

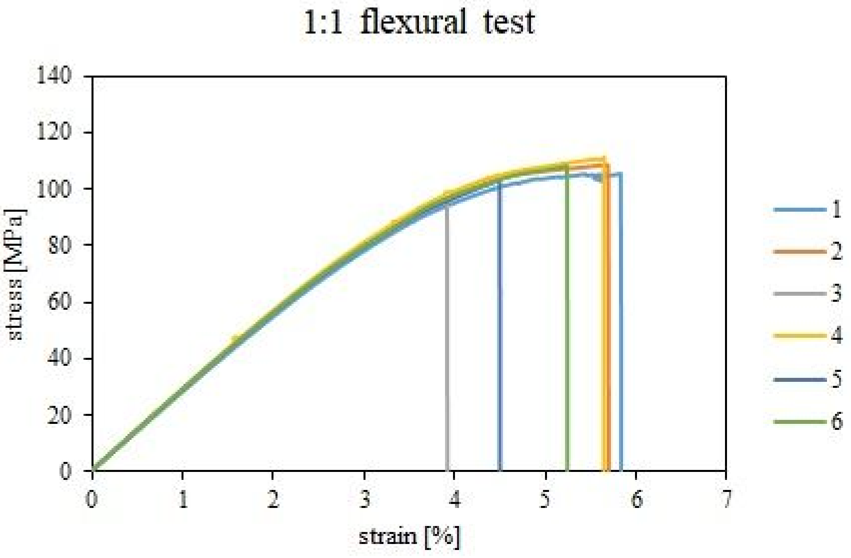

Figure S10. PLA MMA RESIN 1:1 flexural test results.

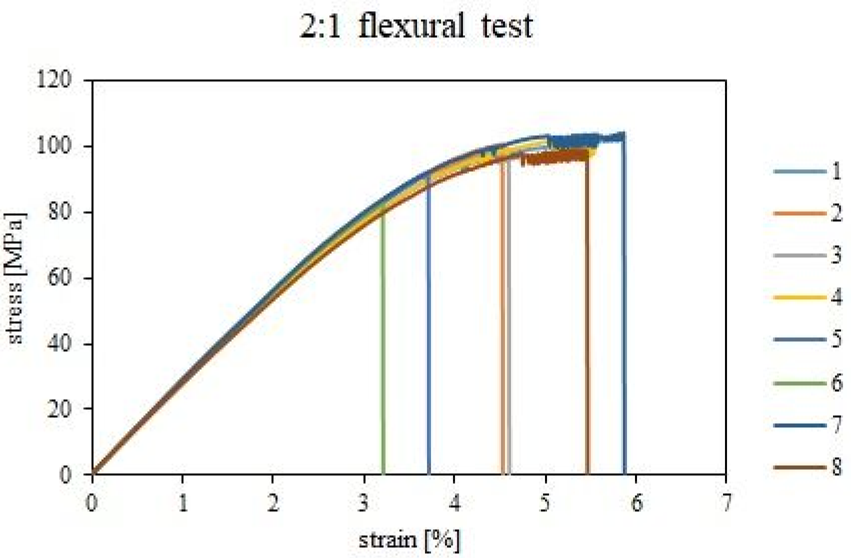

Figure S11. PLA MMA RESIN 2:1 flexural test results.

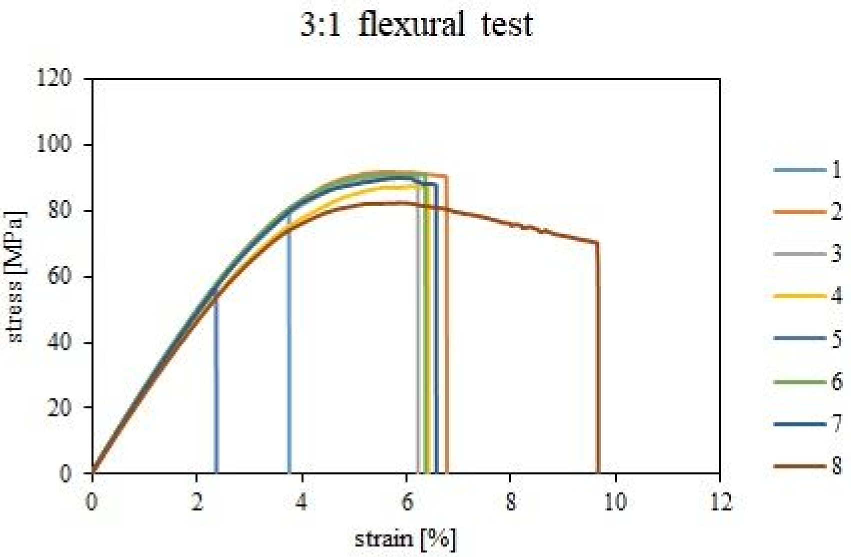

Figure S12. PLA MMA RESIN 3:1 flexural test results.

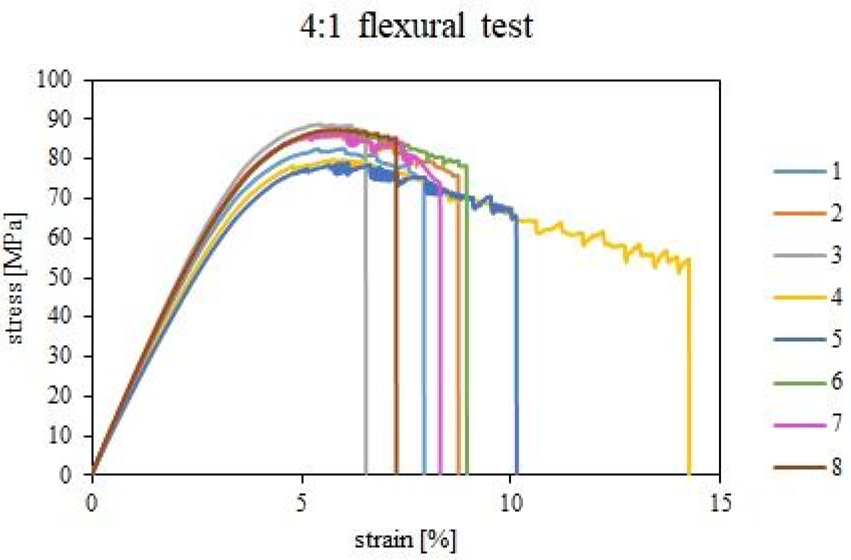

Figure S13. PLA MMA RESIN 4:1 flexural test results.
